# Pan-cancer analysis reveals netrin-1 receptors as potential tumor biomarkers and immune-related therapeutic targets

**DOI:** 10.1038/s41598-025-28437-0

**Published:** 2025-12-17

**Authors:** Ying Gao, Yuchen Hu, Yuyan Zhu, Xiaoli Gao, Wenjun Hao, Xi Chen, Zhendong Zheng

**Affiliations:** 1 Department of Oncology, General Hospital of Northern Theater Command, Shenyang, 110016 Liaoning Province China; 2 Department of Urology, The First Hospital of China Medical University, Shenyang, 110001 China; 3 Department of Urology, The First Affiliated Hospital of Dalian Medical University, Dalian, 116011 China; 4 Department of Endocrinology II, Affiliated Central Hospital of Shenyang Medical College, Shenyang, 110020 China

**Keywords:** NTN1 receptors, Pan-cancer analysis, Tumor biomarkers, Therapeutic targets, Immunotherapy, Cancer, Immunology

## Abstract

The signal pathways mediated by axon guiding molecule netrin-1 (NTN1) are transduced via its several membrane-bound receptors that include deleted in colorectal cancer (DCC), UNC5 (A–D), neogenin 1 (Neo1), melanoma cell adhesion molecule (MCAM), and Down syndrome cell adhesion molecule (DSCAM). Most of these genes play a role in the occurrence and progression of some solid tumors. Sufficient systematic studies have not been performed on the expression characteristics of the role of NTN1 and its receptors in the context of pan-cancer. Based on data from 10,437 subjects with 33 types of solid tumors in The Cancer Genome Atlas, we systematically analyzed the tumor molecular biological characteristics of NTN1 and its receptors through bioinformatics. Candidate small-molecule drugs were identified based on molecular docking analysis. Netrin-1 and its receptors exhibited stereotypical genetic alterations in tumor-suppressor genes or oncogenes. Promoter methylation and miRNA-mediated post-transcriptional inhibition likely represent the primary regulatory mechanisms, whereas the promotion of epithelial-mesenchymal transition (EMT) emerges as a conserved cellular function. we predicted potential small-molecule drugs that could bind to Netrin1 receptors. NTN1 and its receptors can be used as potential targets for tumor immunotherapy. Our results showed the important cancer biological functions of NTN1 and its receptors and their transformational value as candidate tumor biomarkers. This study also showed some critical potential immune-related therapeutic targets and provided a basis for future studies on their role in clinical immunotherapy.

**Supplementary Information:**

The online version contains supplementary material available at 10.1038/s41598-025-28437-0.

## Introduction

Netrins are a family of secretory proteins that play a role in cell migration and axon guidance during embryonic development^1^. Netrin-1 is the most widely studied member of Netrins. It binds to its corresponding receptors and regulates the development of the nervous system, including axonal guidance, cell migration, tumorigenesis, and progression^2^. In recent years, the expression patterns and biological functions of netrin-1 and its receptors have garnered considerable attention.

Netrin-1 receptors, including deleted in colorectal cancer (DCC), UNC5, neogenin 1 (NEO1), Down syndrome cell adhesion molecule (DSCAM), and melanoma cell adhesion molecule (MCAM), participate in the occurrence and progression of some solid tumors^2^. DCC was initially assumed to be a tumor marker for predicting prognosis. However, later it was found that the NTN1 receptor plays an important role in regulating tumor apoptosis, nerve axon guidance, and other cellular processes^3,4^. In the early 1980s, UNC5 was initially characterized as an axon-directed cell surface receptor. UNC5 receptor family proteins contain the death domain (DEATH) homologous to the death domain of tumor necrosis factor^5^. Expression of the UNC5 receptor family is downregulated in many solid tumors such as ovarian cancer, breast cancer, uterine cancer, and colorectal cancer^6^. The DCC and UNC-5 receptor families belong to the dependent receptor family^7^. They induce positive signals, such as cell survival, proliferation, and differentiation in the presence of NTN1, and trigger apoptosis in the absence of NTN1^8^. A gene homologous to DCC^9^, NEO1, belongs to the immunoglobulin (Ig) superfamily protein similar to DCC and UNC5^10^. NEO1 plays a role in many physiological and pathological functions, including cell proliferation, differentiation, and migration^11^. NEO1 also mediates the regulation of solid tumors such as colorectal cancer and gastric cancer. Down syndrome cell adhesion molecule (DSCAM) and CD146 (also known as melanoma cell adhesion molecule [MCAM]) are also netrin-1 receptors. They belong to the immunoglobulin superfamily^12^. DSCAM is a candidate gene associated with the phenotype of Down syndrome. It is expressed in the commissural axons of the spinal cord and binds to netrin-1, which is important for the growth of the commissural axons to the midline and across the midline^12^. Melanoma cell adhesion molecule (MCAM) (CD146) was first identified as a melanoma tumor marker in 1987^13^. Many previous studies reported the role of MCAM in tumors, and evidence exists that it can be used as a potential therapeutic target. Even though many studies reported the presence of the netrin1 receptors in tumors, a lack of systematic study of its expression characteristics, roles, and regulatory mechanisms in the context of pan-cancer exists.

Therefore, in the present study, we used a tumor genomic map (Cancer Genome Atlas, TCGA) and computational biology methods and systematically excavated and analyzed the tumor molecular biological characteristics of netrin1 and its receptors (DCC, UNC5, NEO1, DSCAM, and MCAM). We showed their key genetic changes, co-operative regulatory mechanism, common signal pathway, and immune correlation. We also revealed the key biological functions of netrin1 and its receptors in cancer and their transformational value as the candidate tumor markers and potential targets for tumor immunotherapy.

## Materials and methods

### Mutation analysis

We obtained the structural data of Netrin1 and its receptor from Uniprot (https://www.uniprot.org/), and then downloaded the TCGA mutation data through Cbioportal (http://www.cbioportal.org/). In order to study the non-synonymous mutations in the gene coding region, we removed silencers, splicing regions, and other mutations. Then, we defined the Frame Shift Del, Nonsense_Mutation, Frame Shift Del, Frame Shift Ins, and Splice site as truncated mutations. We then used TCGAAA (http://52.25.87.215/TCGAA/) to assign the TCGA patients into four race groups: African American (AA), Native American (NA), East Asian American (EAA), and European American (EA). Moreover, we applied the best VEST3 and REVEL algorithms in VarCards (http://varcards.biols.ac.cn/) to screen the abovementioned patients for harmful mutations. We employed GraphPad Prism 8 and TBtools for statistical analysis. The data on the function and structural importance of protein sequences were downloaded from UET (http://mammoth.bcm.tmc.edu/uet/), and set the coverage rate < 0.1 as the parameter. The mutation information of Netrin1 and its receptors can be downloaded from CCLE (https://portals.broadinstitute.org/ccle/about) OncoPrinter (http://www.cbioportal.org/oncoprinter) and Mutation Mapper (http://www.cbioportal.org/.

mutation_mapper) were used to visualize the mutation sites. The protein 3D structure data was sourced from the Swissmodel (https://swissmodel.expasy.org/) database and the protein modification sites were derived from the dbPTM (http://dbptm.mbc.nctu.edu.tw/) database.

### Fusion gene analysis

The fusion gene data of Netrin1 and its receptors were derived from the TCGA fusion gene database (http://www.tumorfusions.org/) (Supplementary Data 2). The database included fusion genes predicted by the PRADA analysis of RNA sequencing data of 33 TCGA cancers. We selected tie1 and tie2 as high-confidence outcomes for subsequent analysis. Furthermore, we drew the details of the fusion gene in accordance with the relevant information of NTN1 and its receptors obtained from ENSEMBL (http://asia.ensembl.org/) and CCDS (https://www.ncbi.nlm.nih.gov/CCDS) database.

### Expression and clinical analysis

**Fig. 1:**
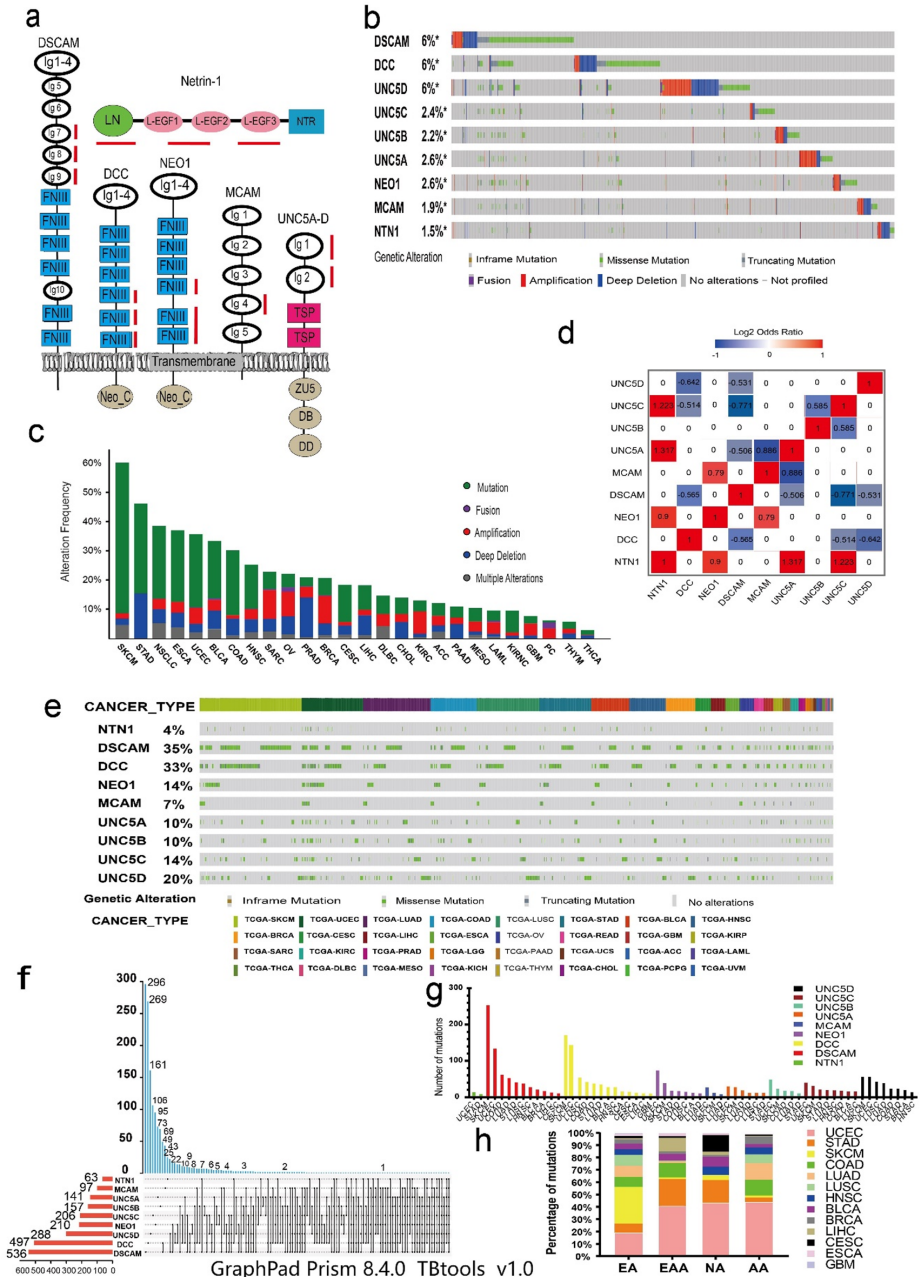
The structures of Netrin1 and its receptors and their genomic changes in pan-cancer. (a) The structures of Netrin1 and its receptors. LN: laminin-like domain; L-EGF1-3: Laminin EGF-like epidermal growth factor repeat (EGF1, EGF2, and EGF3); NTR: c-terminal netrin-like domain). Ig: immunoglobulin, FNIII: fibronectin type-III domain; TSP: thrombospondin type-1 (TSP-1) domain, ZU5: Zona occlucudens 5 (ZU5) domain, DB: DCC-binding domain; DD: death domain. The red underlined part is an important binding part. (b) Study on genomic changes of Netrin1 and its receptors. (c) Statistical analysis of the genomic changes of Netrin1 and its receptors in pan-cancer. (d) Co-occurrence and mutual exclusion of Netrin1 and its receptor mutations; (e) Netrin1 and its receptors have non-synonymous mutations in the coding region in 32 types of The Cancer Genome Atlas (TCGA) cancers. Each of the grey vertical bars represents a patient. (f) The intersection of Netrin1 and its receptor mutations in individuals. (g) The number of mutations of Netrin1 and its receptors in various cancers. Cancer species with > 10 mutations in each member are selected in the chart. (h) The percentage map of the distribution of Netrin1 and its receptor mutations (the number of mutations not < 10) in different races, AA-African American, NA-Native American, EAA-East Asian American, EA-European American(GraphPad Prism 8.4.0 and TBtools v1.0).

The expression data of Netrin1 and its receptors were derived from GSCA (http://bioinfo.life.hust.edu.cn/web/GSCALite/) database. The relevant survival curves were downloaded from Kaplan–Meier Plotter (http://kmplot.com/analysis/). All Kaplan-Meier analyses use the median gene expression as the group tangent, high expression group = expression level > median, low expression group = expression level ≤ median. The clinical data was sourced from LinkedOmics (http://www.linkedomics.org/Login.php) database.Through Lasso-Cox analysis, UNC5B, UNC5D, and NEO1 were determined to be independent prognostic factors, and the PI calculation formula was: PI = (-0.015 × UNC5B expression) + (-0.153 × UNC5D expression) + (-0.205 × NEO1 expression). Covariate inclusion: Forest plots (Fig. 1g) show that the model includes riskScore, age, stage, T stage, N stage, and M stage, suggesting that gene prognosis scores and clinical pathology characteristics should be integrated as covariates into the multivariate Cox model to evaluate the independent effects of each factor on survival. P-value criterion for univariate Cox screening: *p* < 0.1 .Survival analysis Kaplan-Meier Plotter and GSCA are divided into median split, and clinical correlation analysis (LinkedOmics) is divided into median split.

### Methylation and clinical analysis

We used the GSCALite data (http://bioinfo.life.hust.edu.cn) to download the methylation-related bubble chart, which revealed differential methylation of Netrin1 and its receptors among 33 types of TCGA cancers and the corresponding normal tissues. GSCALite was used to obtain methylation beta values for TCGA cancers, excluding samples and sex chromosome probes with detection rates of < 90%. After normalization by BMIQ, the results were screened for tumors vs. normal tissues| Δβ| Probes with ≥ 0.2 and FDR < 0.05. It was clear that FDR (False Discovery Rate) correction was a multiple comparison design for “multiple cancer species and multiple CpG sites”, which could effectively control Type I errors in pan-cancer analysis. Using the data of GSCALite (http://bioinfo.life.hust.edu.cn), we then determined the correlation between the gene expression and methylation of Netrin1 and its receptors in TCGA cancer. The methylation and clinical data were derived from the LinkedOmics (http://www.linkedomics.org/login.php). Spearman correlation between methylation and pathological stage was analyzed in LinkedOmics (|r| ≥0.3, *p* < 0.05. We used the SMART (http://www.bioinfo-zs.com/smartapp/) to conduct detailed analysis of the related methylation rate. Using the SMART tool to locate the UNC5D CpG islandprobe, it must be located in the island coast/shelf area and have a tumor methylation rate greater than 20% in normal tissue.

### Transcription and epigenetics analysis

We downloaded the data of transcription factors and chromatin remodeling factors from CHIPBASE V2.0 (http://rna.sysu.edu.cn/chipbase/) with the binding sites positioned within 1-kb upstream or downstream of Netrin1 and its receptors. The co-expression of each factor with the members of the Netrin1 and its receptors was evaluated in TCGA cancer. We considered that the absolute value was > 0.2 and that the correlation of *p* < 0.05 was a statistically significant critical value. We accordingly downloaded the miRNA data and the location of the potential binding sites on the 3’UTR of Netrin1 and its receptors from miRWalk(http://mirwalk.umm.uni-heidelberg.de/). The experimentally based miRNAs that bind to the Netrin1 and its receptors were queried from STARBASE v3.0 (http://starbase.sysu.edu.cn/). After comparing the predicted mirRNAs in miRWalk and STARBASE v3.0, 30 shared mirRNAs were shortlisted. In STARBASE v3.0, the correlation between the expression of pan-carcer and that of Netrin1 and its receptors was investigated.

### Drug and pathway analysis

We downloaded the correlational network diagram of Netrin1 and its receptors from STRING (https://string-db.org/). GSCALite (https://bioinfo.life.hust.edu.cn/web/GSCALite/) was used to download the overall percentage map and the heatmap percentage map of Netrin1 and its receptors member genes in 10 cancer-related pathways. All data on Netrin1 and its receptor-related drugs were downloaded from PharmacoDB (https://pharmacodb.pmgenomics.ca/), and the relevant absolute value > 0.1 was selected, with *p* < 0.05 serving as meaningful data (Supplementary Data 3).

### Molecular docking simulation

We docked the target protein structures of NTN1 and its receptors obtained from the PDB database with their related sensitive drugs using Autodock Vina (http://vina.scripps.edu/) and Openbabel to screen potential drugs.

### Immune infiltrative analysis

The relationship between mRNA expression of NTN1 and its receptors and the immune infiltration was determined by tumor immune assessment resources (TIMER) and tumor-immune system interaction database (TISIDB). The immunotherapy score data was downloaded from the TCIA (https://tcia.at/) database. The expression and the score of immunotherapy efficacy data were statistically analyzed by the R version 4.1.2.Additional instructions: “For the pan-cancer association analysis between 28 TILs and 9 netrin-1 receptor family genes, FDR < 0.05 was used for multiple comparative adjustments to exclude random associations”.

### Statistical analysis

We performed all heat map analysis and visualization in the Morpheus (https://software.broadinstitute.org/morpheus/). Online analysis websites and software of omicshare (http://www.omicshare.com/tools/) and Cytoscape (v.3.6.1) were also used. “Multiple comparison correction strategy”: It is clear that FDR correction is used in survival analysis (such as multiple cancer species Kaplan-Meier comparison) and gene expression-clinical characteristics association analysis.

Supplementary note: All genomic data in this study are derived from the TCGA single database. Standardized data (such as FPKM standardization of mRNA-seq and beta correction of methylation) that are officially preprocessed by this database are used, and no additional cross-data source batch effect correction is required; For reference data such as protein structure and modification sites, standard formats annotated in the database are used to ensure data consistency.

## Results

### The structure of Netrin1 and its receptors and their genomic changes in pan-cancer

**Fig. 2:**
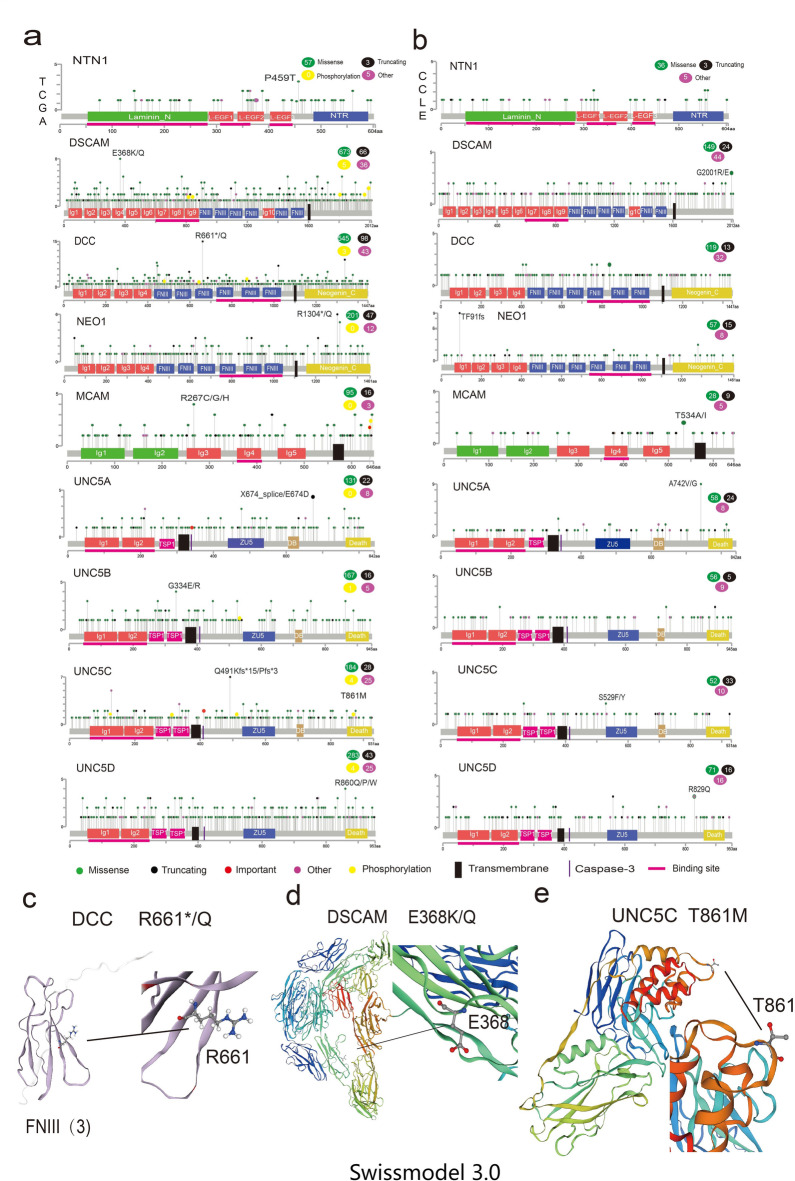
Mutations of NTN1 and its receptors in pan-cancer and the 3D structure of important mutation sites. (a) The amino acid mutation of Netrin1 and its receptors in 32 types of TCGA cancer. The hot spot mutation is marked (Please check the edit and indicate how is the hot spot mutation marked, i.e., with what symbol or color). The substitution mutation is represented by a single-letter amino acid code separated by a slash. Other represents an important mutation site showing damaging in both VEST3 and REVEL algorithms and coverage < 0.1 in the functional and structural importance of protein-sequence positions. Phosphorylation representing the coincident site of phosphorylation and mutation. Transmembrane represents the transmembrane region. Caspase-3 represents the area that can be split by Caspase-3. (b) Binding site represents the amino acid mutation of Netrin and its receptor in CCLE, an important region that binds to Netrin1. Other (Please elaborate for clarity) indicates that it is mutated at both the CCLE and TCGA sites. (c) DCC hot spot mutation R661 mutation Q DSCAM 3D structure. (d) DSCAM hot spot mutation E368K/Q 3D structure. (e) The three-dimensional structure of the T861M mutation site of UNC5C (Swissmodel 3.0).

Based on Uniprot^14,15^and related literature data^16^, we constructed the structure of netrin1 and its receptors and marked the critical binding sites (Fig. 2a). The primary binding sites of NTN1 on DSCAM are on its Ig (immunoglobulin) 7–9^12^ domains and the binding sites on DCC and NEO1 are mainly in their 4th − 6th FNIII (fibronectin type III) domains^17,18^. Similarly, the binding sites of MCAM are in the Ig4, and on UNC5A-D receptors are in the Ig1 and Ig2 domains^19^, in which the Ig1 domain is more important.

We selected data from 10,437 patients from the cancer genome atlas (TCGA) Pan-Cancer Atlas with mutation information from the cBioPortal website^20,21^ for pan-cancer mutation analysis. First, we determined the prevalence of netrin1 and its receptor gene changes in different cancers on the basis of the data extracted from the integrated cell mutations and TCGA copy number changes (Fig. 2b). The overall average mutation frequency of the netrin1 genes and their receptors ranged from 1.5% to 6%. Among these, DSCAM, DCC, and UNC5D had the highest frequencies (6%), and NTN1 had the lowest frequency (1.5%). We then classified the mutation data and constructed a column chart of mutation percentage (Fig. 2c). The mutation rate was the highest in skin cutaneous melanoma (SKCM) (60.14%), which followed by stomach adenocarcinoma (STAD) (46.15%), non-small cell lung cancer (38.46%), esophageal carcinoma (ESCA) (36.96%), and uterine corpus endometrial carcinoma (UCEC) (35.67%). The change was primarily due to gene mutation; however, some subsequent cancers were primarily due to copy number variations (CNV).

We downloaded the correlations between the expression of netrin1 and its receptor CNV and mRNA from the Gene Set Cancer Analysis (GSCA) website^22^ (Supplementary Fig. 1). In many types of cancer, the CNV of netrin1 and its receptors were positively correlated with mRNA expression, which suggested that CNV positively correlates with the expression of netrin1 and its receptors in pan-cancer.

We obtained the mutation information from 10,437 patients in the TCGA PanCancerAtlas from cBioPortal and analyzed the co-occurrence and mutual exclusion of pan-cancer mutations after screening. NTN1 had a co-occurrence relationship with UNC5A, UNC5C, and NEO1 (*p* < 0.05). MCAM had a co-occurrence relationship with NEO1 and UNC5A. UNC5B had a co-occurrence relationship with UNC5C. DSCAM had a mutually exclusive relationship with UNC5A, UNC5C, and UNC5D (*p* < 0.05), DCC had a mutual exclusivity relationship with UNC5C and UNC5D (Fig. 2d). We further collated the mutation information regarding netrin1 and its receptors (Fig. 2e). SKCM had the most mutations of all cancers, which was followed by UCEC, lung adenocarcinoma (LUAD), colon cancer, STAD, and BLCA. Only one case of NTN1 and its receptor mutation was found in pheochromocytoma, paraganglioma, and uveal melanoma.

We screened the non-synonymous mutations (Fig. 2f). NTN1 had 63 patients with complete mutation information (65 mutations). Others as follow : DSCAM(536/763), DCC(497/665), NEO(210/252), MCAM (97/111), UNC5A(141/157), UNC5B(157/181), UNC5C(206/231), UNC5D(288/337). We selected nine genes of one patient (TCGA-AP-A1D V-01; Patient ID in TCGA) using crossover screening, and the cancer types were UCEC (15), colon adenocarcinoma (COAD) (1), and SKCM (1). Three patients had mutations in eight genes, of which TCGA-A5-A0G1-01 had mutations in genes except for DSCAM; and all cancers were UCEC (8). Patient TCGA-IB-7651-01 had mutations in genes except for NTN1. Cancers were pancreatic adenocarcinoma (PAAD) (14) and SKCM (2). Patient TCGA-AP-A059-01 had mutations in genes except for NTN1. The types of cancer were UCEC (11), lung squamous cell carcinoma (LUSC) (1), and ESCA (1). These mutations were common in DCC, NEO1, MCAM, and UNC5A-D.

We selected the cancer data that had no fewer than 10 mutations in netrin1 and its receptors to construct a histogram (Fig. 2g). We found that SKCM and UCEC ranked among the top two mutant cancers for netrin1 and its receptors, of which DSCAM had the most mutations in SKCM, followed by DCC. Based on the ethnical distribution (Fig. 2h), the the greatest number of cancer mutations among the four races were UCEC and SKCM, primarily in European-Americans. STAD was primarily found in East Asian-Americans and Native Americans. COAD was primarily found in East Asian Americans and African Americans. Lung cancer (LUAD and LUSC) was primarily found in European-Americans and African-Americans.

### Mutations of NTN1 and its receptors in pan-cancer and the 3D structure of important mutation sites

**Fig. 3:**
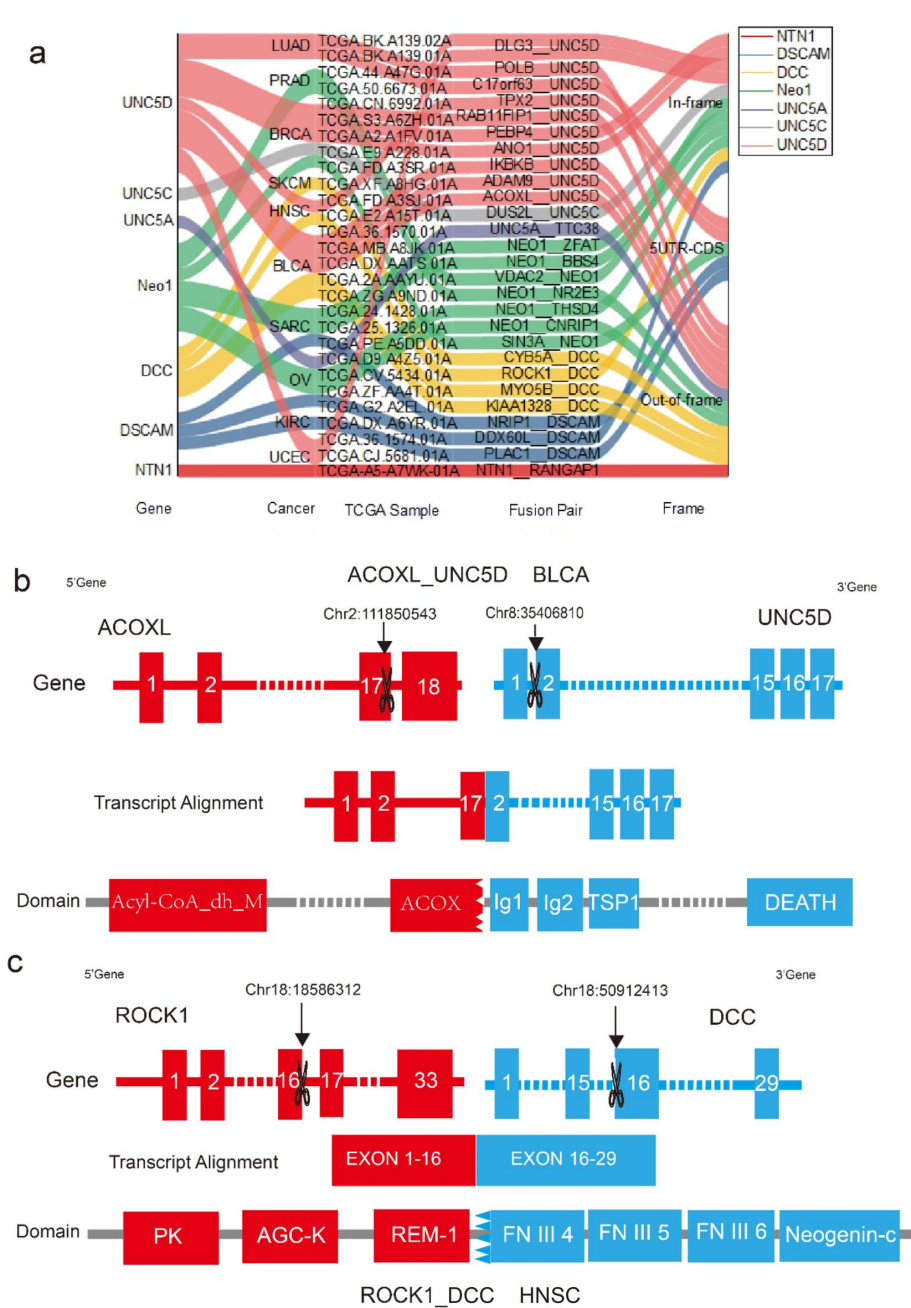
Fusion genes of Netrin1 and its receptors. (a) The fusion genes of NTN1, DSCAM, DCC, NEO1, UNC5A, UNC5C, and UNC5D in various cancers. (b) The process of fusion transcription of ACOXL and UNC5D in BLCA. Red indicates exon of *ANO1*, blue indicates exon of *UNC5D*, and dotted line indicates the connection of two partial genes. (c) The process of fusion transcription of ROCK1 and DCC in HNSC. Red indicates exon of *ROCK1*, blue indicates exon of *DCC*, and dotted line indicates the connection of two partial genes.

We further analyzed the hot spot mutations (Supplementary data 1) of these nine genes (Fig. 3a) using VEST3 and REVEL algorithms and coverage of < 0.1 in the functional and structural importance of protein sequence positions as screening conditions^23^. These 15 hot spot mutations did not fulfill the criteria of displaying dual damage in VEST3 and REVEL algorithms and coverage of < 0.1 in the functional and structural importance of protein sequence positions. However, one of the hot spot mutations in DSCAM, E368K (Fig. 3d), occurred in seven patients with two cancers (SKCM, UCEC) and was damaged in both VEST3 and REVEL algorithms. The hot spot mutation R661*/Q of DCC (Fig. 3c) occurred in 8 patients with UCEC out of 15 patients with 6 cancers (OV, SKCM, BRCA, COAD, BLCA, and UCEC). Among the 15 patients, 13 patients with R661* mutation had truncated mutation. Of 15 patients, eight patients had UCEC and 13 R661* mutations, where one of the hot spot mutations was truncated mutation. The mutation R1304* of NEO1 occurred in five patients with three cancers (UCEC, rectum adenocarcinoma [READ], and COAD), which were truncated mutations. One of the hot spot mutations of UNC5A (X674_splice) was found in three patients with three cancers (UCEC, kidney renal clear cell carcinoma [KIRC], and kidney renal papillary cell carcinoma [KIRP]). The hot spot mutations belonged to truncated mutation UNC5B (G334E/R), which occurred in four patients with SKCAM. The hot spot mutations Q491Kfs*15/Pfs*3 of UNC5C, which were both damaged in VEST3 and REVEL algorithms, occurred in seven patients with five cancers (STAD, UCEC, ACC, CESC, and ESCA), all of which were truncated mutations. The hot spot mutation P459T of NTN1 occurred in three patients with two cancers (STAD and READ), and the hot spot mutation R267C/G/H of MCAM occurred in four cancers (STAD, BLCA, UCEC, and LUSC). The three-dimensional structure (Fig. 3c and d, and 2e) of the hot spot mutation R661*/Q of DCC, the hot spot mutation E368K/Q of DSCAM, and the mutation site, T861M of UNC5C, was obtained from the Swiss model database^24–26^.

Based on the dbPTM database^27^, we further analyzed the protein modification sites and compared them with the mutant sites. We found that two phosphorylation sites, S848Y (UCEC) and S860F (SKCM), were present, which were coincident with the mutation sites on the Ig9 domain (important binding domain) of DSCAM. The Y881* (LUAD) mutation site of the 5th FNIII domain (important binding domain) of DCC coincided with the phosphorylation site. Y881C (UCEC) was found to be double damaged in VEST3 and REVEL algorithms. Y641* (UCEC) with coverage of < 0.1 was found in the functional and structural important protein sequence position and acetylation and ubiquitin modification site overlapped (red marked site) in the 640th site next to truncated mutation. S531Y (SKCM) of UNC5B and S127L (SKCM) of Ig1 domain (important binding domain) of UNC5C all overlapped with their phosphorylation sites. T861M phosphorylation coincided with the mutation site on the DEATH structure of UNC5C, which was double damaged in the VEST3 and REVEL algorithms.

We tagged the caspase-3 cleavage region based on the Uniprot database. In UNC5A, we found that the S341L (UCEC) mutation site (red marked site) was on its caspase-3 cleavage region, which was double damaged in VEST3 and REVEL algorithms. We found that the S417L (UCEC) mutation site (red marked site) was near its caspase-3 cleavage region (415, 416) and was damaged in both VEST3 and REVEL algorithms.

Overall, DSCAM had the maximum mutation sites (in almost every region), followed by DCC. UNC5A-D key mutations were distributed in and around the important domains such as Ig, ZU5, and DEATH. Regardless of the hot spot mutant cancer species or the coincidence of the modified sites with mutation sites, SKCM and UCEC were relatively large, corresponding to overall mutations (Fig. 2e). The mutation distribution of NTN1 and its receptor members in CCLE (Cancer Cell Line Encyclopedia) was consistent with the TCGA data (Fig. 3b). These important mutation sites can be an important topic for future research.

### Fusion gene of Netrin1 and its receptors

**Fig. 4:**
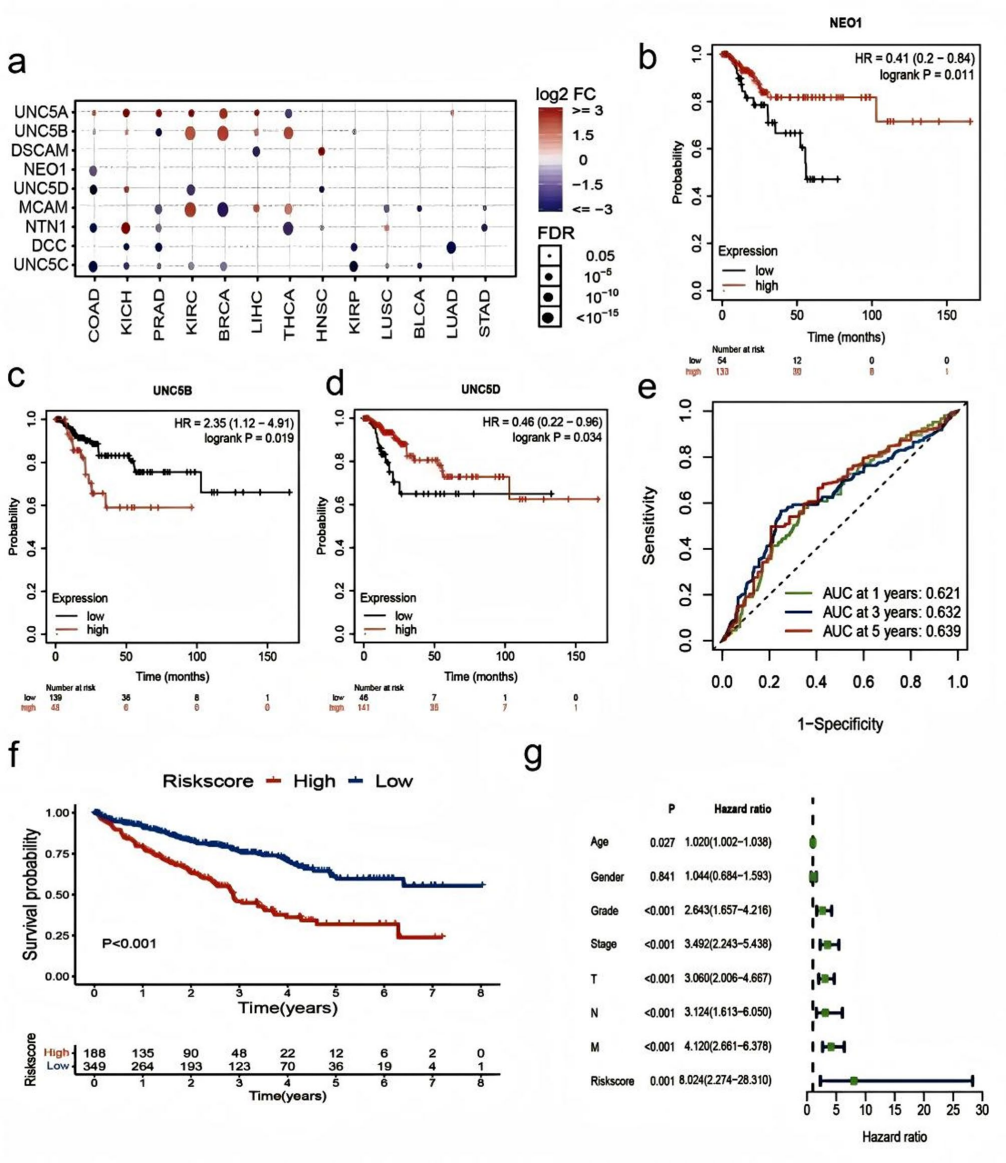
Analysis of survival and prognosis model. (a) The difference in the expression of Netrin1 and its receptor in different cancers of TCGA and their normal tissues. Colors from purple to red represent multiple changes between tumors and normal tumors (please check the highlighted text. Specify what is the former tumor type). Point size implies importance. The dot was filtered by the fold change (FC > 2) and significance [false discovery rate) FDR < 0.05]. (b) The OS survival curve of NEO1 in bladder urothelial carcinoma (BLCA). (c) The OS survival curve of UNC5B in BLCA. (d) The overall survival (OS) survival curve of UNC5D in BLCA. (e) Area under the curve (AUC) of ROC curves displaying the predictive accuracy of the risk scores in the training and validation cohorts.(f) Kaplan-Meier analysis of the prognostic model in the training and validation cohorts. (g) Comparison of HR values of different factor models.

We predicted the tie1 and tie2 fusion transcript of NTN1 and its receptor from the TumorFusions Database^28^ in TCGA cancers (Fig. 4a) (Supplementary data 2). NTN1 only predicted the NTN1-RANGAP1 and NEO1 fusion gene in endocrine-associated cancer (OV, BRCA, and prostate adenocarcinoma [PRAD]) in UCEC. Six of the 11 predicted UNC5D fusion genes were destroyed at chr8:35406810, which included three in BLCA, two in BRCA, and one in head and neck squamous cell carcinoma (HNSC). The UNC5D fusion transcription Acoxl-UNC5D was predicted in BLCA and ACOX (acyl CoA [coenzyme A] oxidase) that may induce the tumorigenic conversion of rat urothelial carcinoma^29^. UNC5D may serve as a new inhibitor of bladder cancer through the UNC5D/DAPK pathway^30^. The bladder cancer data set showed that the expression of the ACOX1 gene was significantly associated with the expression of DAPK1. Knockout of DAPK1 in the T24 cells of bladder cancer downregulated the expression of ACOX1. We constructed a detailed map of the fusion gene according to the ENSEMBL (a genome browser for vertebrate genomes)^31^ and the relevant information from the consensus coding sequence database in the National Center for Biotechnology Information(NCBI)^32^. We found that ACOXL formed a fusion gene with UNC5D by destroying the ACOX domain(Fig. 4b), whereas the domains of UNC5D were preserved. ACOX domain is a shared domain of the ACOX family. This suggested that the role of UNC5D in bladder cancer may be associated with the ACOX family.

**Fig. 5:**
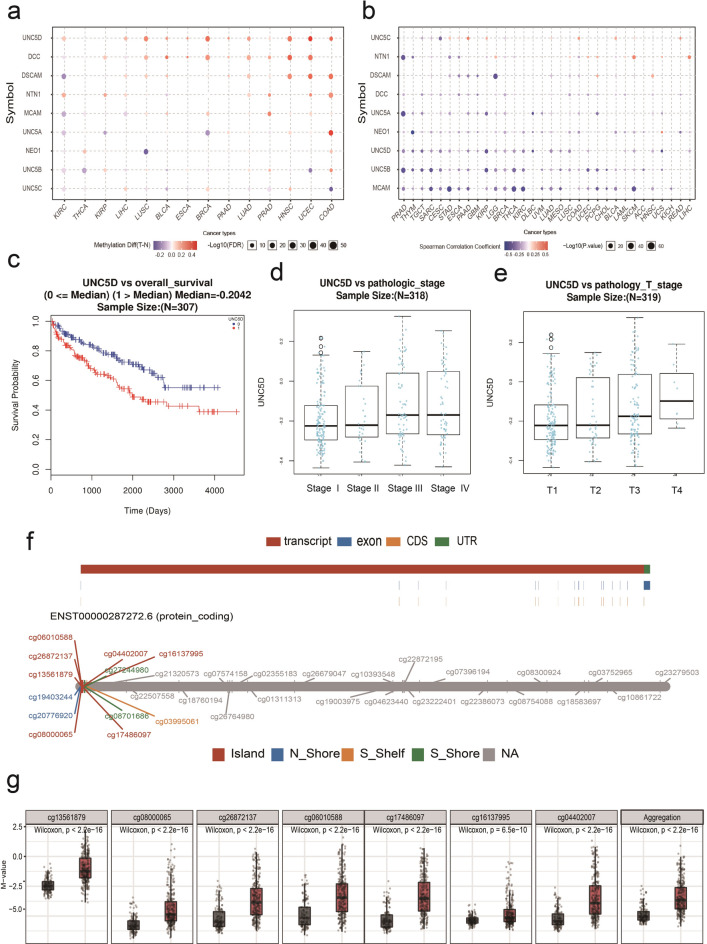
Methylation analysis of Netrin1 and its receptors. (a) The difference of Netrin1 and its receptor genes in methylation bubble map between TCGA cancer and normal samples. The blue dot represents the downregulation of methylation in the tumor, and the red dot represents the upregulation of methylation in the tumor. The darker the color, the greater the difference. The size of the dot represents the statistical significance. The larger the dot, the more significant it is. (b) The relationship between methylation and the expression of Netrin1 and its receptor genes in TCGA cancer. The blue dot indicates that the level of gene methylation was upregulated and the gene expression was downregulated. The red dot indicates that the level of gene methylation was upregulated and the gene expression was also upregulated. The darker the color, the higher the correlation. The size of the point represents the statistical significance; the larger the size, the more significant it is. (c) UNC5D methylation and survival graph. (d) UNC5D methylation and the pathological stage diagram. (e) UNC5D methylation and pathologic T-stage diagram. (f) UNC5D methylation cytosine-phosphate-guanine(CpG) distribution map. (g) 7-CpG methylation box diagrams of the Island.

We predicted the ROCK1-DCC fusion transcripts in HNSC. The expression of Rho-associated protein kinase (ROCK1) was closely associated with the growth and lymph node metastasis of laryngeal squamous cell carcinoma^33,34^. A previous study and Fig. 5a showed that DCC was frequently methylated in HNSC^35^. The upregulation of this methylation may increase the formation of ROCK1-DCC fusion transcripts. The fusion site was in FNIII 4, an essential domain of the interaction of DCC and NTN1 (Fig. 4c), which suggested that DCC may participate in the mechanism underlying HNSC and ROCK1, thereby providing a new concept for elucidating the mechanism underlying HNSC.

UNC5D predicted fusion transcripts ANO1-UNC5D, PEBP4-UNC5D, and RAB11FIP1-UNC.

5D in BRCA, the calcium-activated chloride channel anoctamin 1 (ANO1) was highly-expressed and amplified in many cancers, including breast, pancreatic, and prostate cancers. Downregulation of ANO1 expression and function significantly inhibited cell proliferation, migration, and invasion of many cancer cell lines. ANO1 can act as a transcriptional regulator of HER2 (c-erbB-2), and ANO1 inhibitors could treat BRCA patients who were resistant to HER2 targeted therapy^36^. Based on the LinkedOmics database^37^ expression and clinical correlation analysis, the expression of ANO1 and UNC5D was associated with the PAM50 (Prediction Analysis of Microarray 50) of BRCA. Likely, the fusion was closely associated with the occurrence of BRCA and PAM50 typing.

### The expression and clinical analysis of Netrin1 and its receptors in pan-cancer

We analyzed the differential expression of TCGA cancers using GSCA (Fig. 1a). We found that the expression of NTN1, DCC, NEO1, UNC5C, and UNC5D was low, whereas the expression of UNC5A was high. Differential expression of UNC5B and UNC5C was found in renal cell carcinoma (KIRC, KIRP, and KICH). The expression of UNC5C was low in urinary tumors (KIRC, KIRP, KICH, PRAD, and BLCA).

We further analyzed the survival curve using the gene expression profiling interactive analysis (GEPIA)^38^ and Kaplan–Meier Plotter^39^.We found that low expression of NEO1(Fig. 1b) and UNC5D(Fig. 1d) showed poor prognosis in BLCA, whereas the high expression of UNC5B showed poor prognosis (Fig. 1c). To explore the clinical value of these genes, we used Netrin1 and its receptors involved in Lasso-penalized Cox analysis (Supplementary Fig. 3).The prognostic index (PI)=(-0.015* expression level of UNC5B) + (-0.153* expression level of UNC5D) + (-0.205* expression level of NEO1). The time-dependent ROC curves for OS at 1, 3, and 5 years exhibited good predictive performance with this model (Fig. 1e). The K-M curves showed that patients in the high-risk group indicated a poorer prognosis (Fig. 1f).The forest plot shows that in the nomogram, riskScore, age, stage, T stage, N stage, M stage are the main influencing factors (Fig. 1g). These results suggest that the nomogram with risk scores can be used as an effective method to predict patient prognosis in clinical practice.

In addition, we found that all members of NTN1 and its receptors affected the survival and prognosis of KIRC (Supplementary Fig. 4a-i). NTN1, DCC, NEO1, MCAM, UNC5B, UNC5C, and UNC5D showed low expression is associated with poor prognosis, whereas DSCAM and UNC5A showed the opposite trend. Through further expression-clinical analysis of LinkedOmics, we found that UNC5D was associated with the pathologic stage (Supplementary Fig. 4j) and pathology N stage (Supplementary Fig. 4k). Recovery of UNC5D expression in renal cancer cells significantly inhibited cell proliferation, whereas the knockdown of UNC5D increased cell growth^40^. Low expression of UNC5D is significantly associated with poor prognosis of renal cell carcinoma, and loss of its expression may be a potential concomitant feature or regulatory factor of renal cell carcinoma. The high expression of DSCAM in HNSC implied a poor outcome and its expression was associated with radiation therapy (Supplementary Fig. 4l). After radiotherapy, the expression of DSCAM decreased, which suggested that DSCAM can play a role in HNSC and sensitivity to radiotherapy.

### Methylation analysis of Netrin1 and its receptors

We analyzed the methylation of netrin1 and its receptor genes in TCGA cancers using GSCALite (Fig. 5a). We found that NTN1, DCC, DSCAM, MCAM, and UNC5D were methylated in many cancers, of which UNC5D was the most prominent. The upregulation of methylation was found in all 12 types of cancers. NTN1 showed co-methylation patterns with its receptors in COAD, UCEC, PRAD, LUAD, LUSC, liver cancer (LIHC), KIRP, and KIRC. The co-methylation pattern with UNC5D was the most common cancer species (Fig. 5a). We analyzed the relationship between methylation and the expression of netrin1 and its receptors and found that the methylation of each gene and their expression was negatively correlated; only a few of them were correlated positively (Fig. 5b).

Further methylation and clinical analysis of LinkedOmics revealed that UNC5D showed hypermethylation and poor survival in KIRC (Fig. 5c), findings that correlated with its low expression and adverse prognosis in this cancer.

The methylation of UNC5D in KIRC was associated with the pathologic stage (Fig. 5d) and pathology T stage (Fig. 5e). Methylation was directly proportional to the pathological stage, which was also inversely proportional to the expression of UNC5D. No significant difference was found in the relevant data of KIRP. NEO1 showed hypomethylation and poor survival in KIRC, which was clinically associated with pathologic stage, pathology T stage, and pathology M stage (Supplementary Fig. 2a–d). DSCAM was hypermethylated in UCEC and was associated with the histologic type, with the highest degree of methylation in the endometrioid endometrial adenocarcinoma (Supplementary Fig. 2e).

We analyzed this methylation using the Shiny Methylation Analysis Resource Tool (SMART)^41^. We found 33 CpGs (cytosine, phosphoric acid, and guanine sites) in UNC5D, of which seven were located in island 5, located on the shore and shelf (referring to 2 kb and 4 kb from the edge of CpG Island, with N representing upstream and S representing downstream ) (Fig. 5f). All seven CpGs of the UNC5D CpG island showed upregulation of methylation (Fig. 5g), whereas the others showed the opposite trend. UNC5D in KIRC regulated methylation through CpG island, which affected its transcriptional regulation and expression.

### Transcription and epigenetics analysis of netrin1 and its receptors

**Fig. 6:**
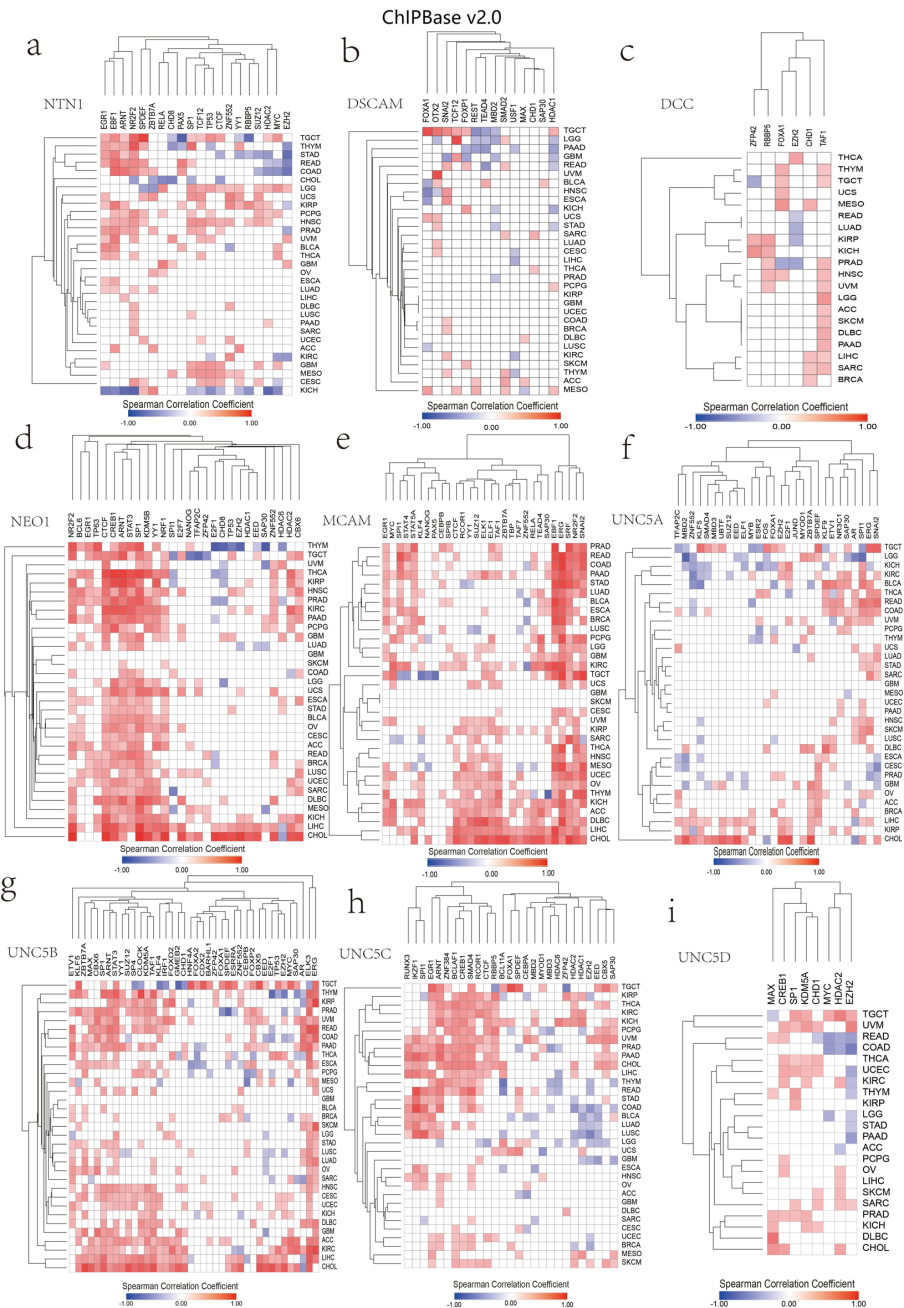
Transcriptional regulators of Netrin1 and its receptors and their co-expression in TCGA cancers(ChIPBase v2.0). The co-expression of transcriptional regulators of (a) NTN1, (b) DSCAM, (c) DCC, (d) NEO1, (e) MCAM, (f) UNC5A, (g) UNC5B, (h) UNC5C, and (i) UNC5D and the genes in TCGA cancers. We selected the absolute value of Spearman Correlation Coefficient to be not < 0.2 as the valid data. Red indicates a positive correlation between the gene and its transcriptional regulatory factors in cancer (*p* < 0.05), while blue indicates the vice versa. The darker the color, the higher is the correlation.

We downloaded the coexpression data of netrin1 and its receptors and transcription factors, which were located within 1-kb upstream and downstream regions, as well as chromatin remodeling factors in TCGA cancers from ChIPBase v2.0^42^ These included 20 NTN1 modifiers (Fig. 6a), 14 DSCAM modifiers (Fig. 6b), 6 DCC modifiers (Fig. 6c), 28 NEO1 modifiers (Fig. 6d), 29 MCAM modifiers (Fig. 6e), 28 UNC5A modifiers (Fig. 6f), 40 UNC5B modifiers (Fig. 6g), 27 UNC5C modifiers (Fig. 6h), and 8 UNC5D modifiers (Fig. 6i).

In terms of transcriptional regulation, forkhead box A1 (FOXA1) downregulates DCC and UNC5B in PRAD. Studies showed that the loss of FOXA1 might play a role in the progression of prostate cancer to neuroendocrine prostate cancer^43^. DCC potentially inhibits prostate cancer progression^44^. UNC5B can be used to predict prostate cancer metastasis^45^. The expressions of UNC5B and DCC are low in PRAD (Fig. 4a), suggesting new concepts for further research on relevant underlying mechanisms. TP53 regulates NTN1, UNC5B, and NEO1. NEO1, a DCC homolog, is regulated by tumor protein p53 (P53) and tumor protein p63 (P63) and is highly sensitive to medications, thereby providing an aspect for future research.

An increased EZH2 expression in many solid tumors is a part of epigenetic regulation and is associated with disease deterioration, transcription silencing, distant metastasis, and the differential suppression of tumors^46^. EZH2 promotes tumorigenesis by altering the expression of several tumor suppressor genes^47^. Furthermore, EZH2 regulates NTN1, DCC, NEO1, and UNC5A-D. Moreover, EZH2 negatively regulates NTN1, NEO1, and UNCB-D in thymoma. Of these, NEO1 has the strongest correlation (*r* = − 0.5814), suggesting that EZH2 may alter the expression of NTN1, NEO1, and UNCB-D to promote the occurrence of thymoma. EZH2 downregulates NTN1 and UNC5B-D, which are less expressed in COAD (Fig. 1a). Studies have shown that EZH2 can be used as a prognostic indicator of colon cancer and can be a therapeutic target for patients with colon cancer in the future^48^. This suggests that NTN1 and UNC5B-D may inhibit the occurrence and progression of COAD via EZH2.

Histone deacetylases (HDACs) are histone-modifying enzymes involved in the pathogenesis of several diseases, especially cancer^49^. HDACs are new anticancer medications. The US Food and Drug Administration (FDA) approved four HDAC inhibitors, namely vorinostat, romidepsin, panobinostat, and belinostat to be included in an anticancer treatment regime^50^. HDAC1 and HDAC2 are necessary for the growth and survival of kidney cancer cells^51^. HDAC1 and HDAC2 are expressed in nearly 60% of renal cell carcinomas^52^. We found that DSCAM was downregulated by HDAC1 in KICH, whereas NTN1 was downregulated by HDAC2 in KICH. In general, NTN1, NEO1, DSCAM, UNC5C, and UNC5D are all regulated by HDAC members in renal cancer, suggesting that NTN1, NEO1, DSCAM, UNC5C, UNC5D, and especially NEO1 and UNC5C may be essential for the regulation and outcome of renal cancer. HDAC inhibitors (HDACis) inhibit proliferation and promote apoptosis in LIHC cells. Yin yang-1 (YY1) reduces the sensitivity of LIHC cells to HDACis and may be a potential therapeutic target for hepatocellular carcinoma^53^. We found that NEO1 was positively regulated by HDAC1, 2, and YY1, whereas was negatively regulated by HDAC6 in LIHC, suggesting that NEO1 may play an important role in the proliferation and apoptosis of liver cancer cells. In colorectal adenocarcinoma, the significantly increased immunohistochemical staining of HDACs, including HDAC1, HDAC2, HDAC3, and HDAC4, was observed^54^. NTN1, NEO1, UNC5C, and UNC5D were all downregulated by HDAC2 and showed low expression in COAD (Fig. 1a), suggesting new research concepts regarding relevant underlying mechanisms. An HDACi has an inhibitory effect on cholangiocarcinoma (CHOL)^55^. We found that NEO1 was upregulated by HDAC1 and HDAC2 in CHOL and downregulated by HDAC6, suggesting that NEO1 may play an important role in CHOL.

**Fig. 7:**
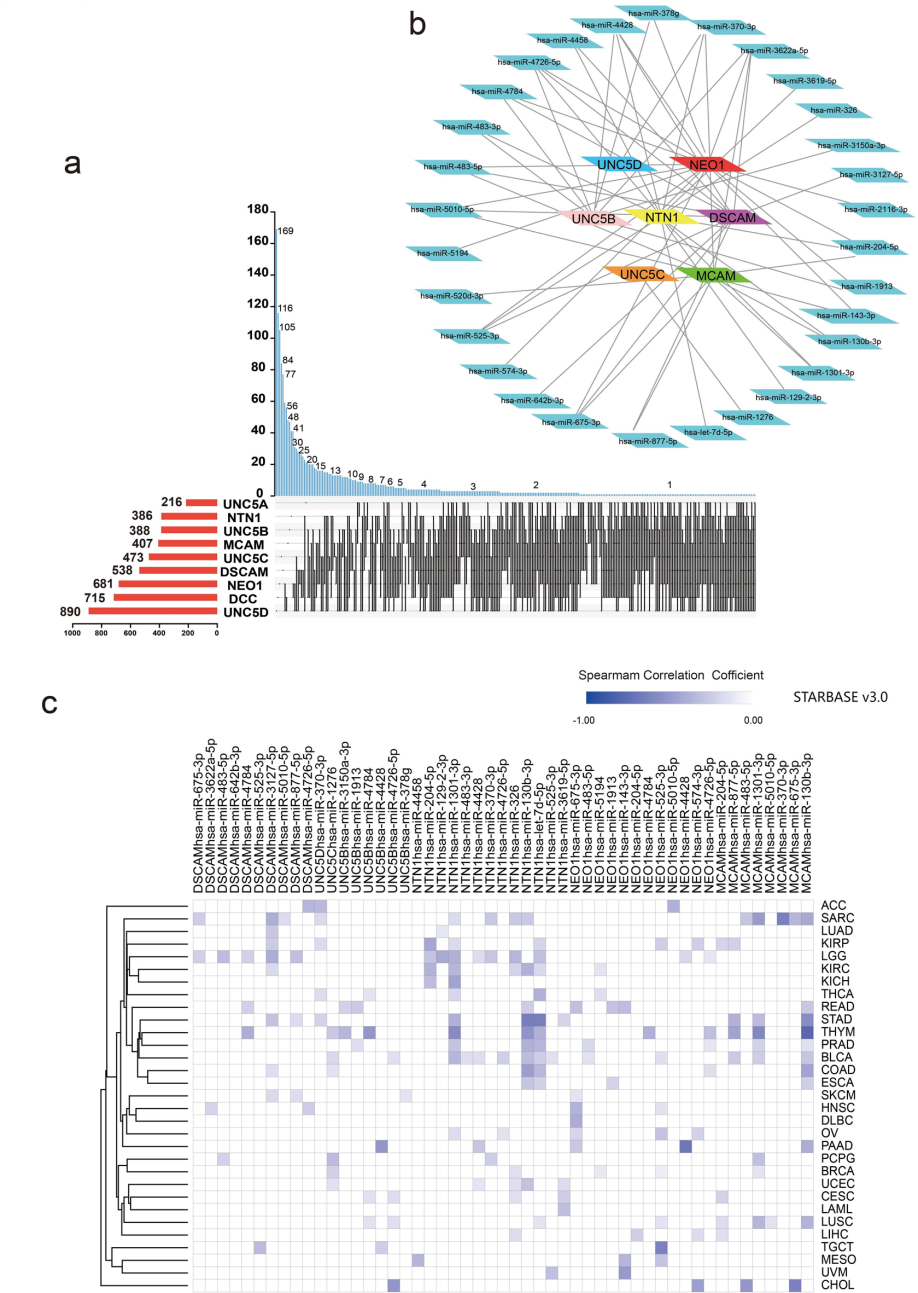
Identification of miRNAs that may target Netrin1 and its receptors. (a) Statistical distribution of miRNAs and Netrin1 and its receptors(STARBASE v3.0). (b) The miRNAs that binds to at least 4 members of Netrin1 and its receptors. (c) The heat map of the correlation between the expression of 30 miRNA in pan-carcinoma and the expression of Netrin1 and its receptors. The darker the color, the higher is the correlation.

Based on the miRWalk database (TarPmiR algorithm)^56^, we predicted a total of 4694 micro RNAs (miRNAs) binding to netrin1 and its receptors at the 3’UTR (untranslated regions) (Fig. 7a). In terms of the total number of binding miRNAs or the total number of miRNAs binding to only one gene, the numbers of UNC5D bindings were the highest, followed by the numbers of DCC and NEO1 bindings. miRNAs with binding numbers not less than 2 accounted for 85.0% of the total. Total 318 miRNAs with binding numbers more than 4 for netrin1 and its receptors were present. Comparing with the data obtained from STARBASE v3.0^57^, we found 30 common miRNAs (Fig. 7b). We analyzed the correlations between the expressions of these miRNAs and the binding of NTN1 and its receptors in TCGA (Fig. 7c). More than 50% of the miRNAs strongly inhibited NTN1 in LGG (low-grade glioma) and BLCA. Half of the miRNAs inhibited DSCAM in LGG. In sarcoma and LUSC, more than 50% of the miRNAs inhibited MCAM. Among these significant results, hsa-miR-130b-3p had the most potent inhibitory effect on MCAM in thymoma (*r* = − 0.574), hsa-miR-4428 had the most potent inhibitory effect on NEO1 in PAAD (*r* = − 0.507), hsa-miR-130b-3p had the most potent inhibitory effect on NTN1 in STAD (*r* = − 0.48), and hsa-miR-4726-5p had the most potent inhibitory effect on UNC5B in CHOL (*r* = − 0.401).

### Drug and pathway analysis of netrin1 and its receptors in pan-cancer

**Fig. 8:**
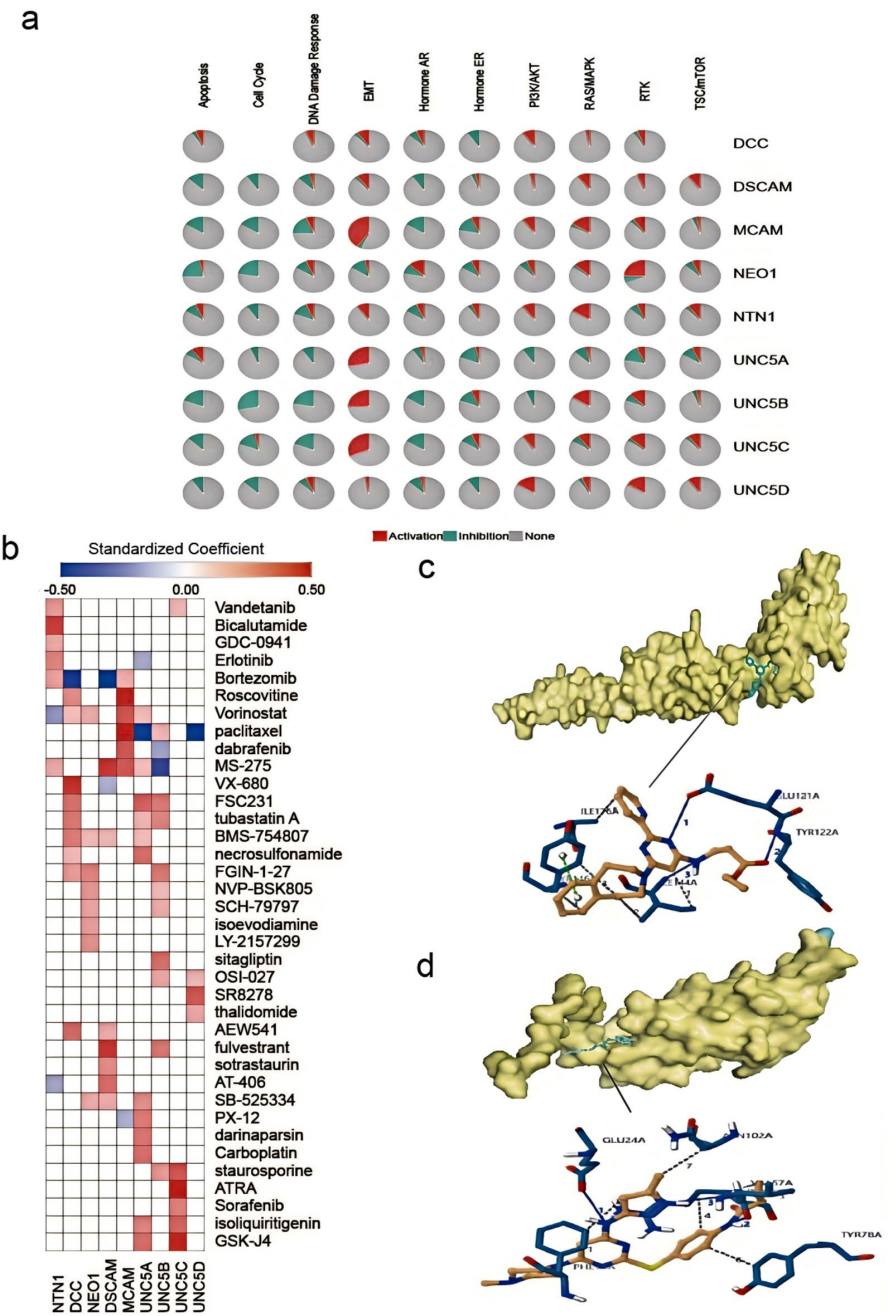
Netrin1 and its receptor pathway and its potential drug prediction. (a) Global percentage indicates the percentage of gene’s function (activation or inhibition) for each pathway in pan-cancer. (b) Drug sensitivity/tolerance heatmap of Netrin1 and its receptors(c)NEO1 docking with VX-680 (d) UNC5A docking with GSK J4.

We analyzed the role of netrin1 and its receptors in the pan-cancer pathway of TCGA using GSCALite (Fig. 8a). We found that all members of the netrin1 family and its receptors inhibited the cell cycle except DCC, and other members of the UNC5C family completely inhibited the cell cycle. UNC5B most strongly inhibited the cell cycle and DNA damage responses. MCAM most strongly activated EMT. UNC5A most strongly inhibited hormone receptors such as estrogen receptor (ER) and receptor tyrosine kinase (RTK). NTN1 and UNC5A-D completely activated EMT, whereas MCAM and UNC5A-C strongly activated EMT. NEO1 most strongly activated RTK.

We downloaded cancer chemotherapy information related to netrin1 and its receptors from the cancer medication database PharmacoDB including 759 compounds from seven databases, namely CCLE, GDSC1000, gCSI, GRAY, FIMM, CTRPv2, and UHNBreast^58^. We selected top-five sensitive chemotherapeutic agents with *p* < 0.05 and standardized coefficient > 0.1 reported in the literature^23^. We removed the data from |standardized coefficient| <0.1 and plotted a graph (Fig. 8b) (Supplementary data 3).

Vandetanib, erlotinib, dabrafenib, and sorafenib are biologically-targeted antineoplastic agents characterized by tyrosine kinase inhibition. The FDA approved tyrosine kinase inhibitors to treat cancers including thyroid cancer, non-small cell lung cancer, melanoma, liver cancer, and renal cell carcinoma. High expressions of NTN1 and UNC5C were highly sensitive to vandetanib, NTN1 was highly sensitive to erlotinib, and UNC5A showed the opposite behavior. MCAM was sensitive to dabrafenib, whereas UNC5B showed the opposite behavior. UNC5C was sensitive to sorafenib. Paclitaxel is a natural secondary metabolite isolated and purified from the bark of *Taxus chinensis var. mairei*. It has sound antitumor effects, especially in ovarian cancer^59^, endometrial cancer^60^, and breast cancer^61^ with high incidences. MCAM and UNC5B with high expression are highly sensitive to paclitaxel. MCAM is the most sensitive (standardized coefficient = 0.59); UNC5A and UNC5D showed strong medication resistance (standardized coefficient = − 0.79, standardized coefficient = − 0.82). BMS-754,807 and AEW541 significantly inhibited cell proliferation in triple-negative breast cancer (*p* < 0.001)^62^. Highly-expressed DCC and DSCAM were highly sensitive to BMS-754,807 and AEW541, and NEO1 and UNC5A were also sensitive to BMS-754,807. Fulvestrant is a selective ER degrader approved for the first- and second-line treatment in postmenopausal women with hormone receptor-positive advanced breast cancer^63^. The highly-expressed DSCAM and UNC5B were highly sensitive to fulvestrant. Bortezomib is an essential part of antimyeloma therapy. It has good clinical efficacy and controllable side effects. Highly-expressed NTN1 and MCAM are sensitive to bortezomib, as opposed to DCC and DSCAM. Studies showed that DCC enhanced sensitivity compared with bortezomib^64^. MS-275, tubastatin A, and vorinostat are all HDACis. They were approved as anticancer medications by the FDA. They act as transcriptional inhibitors by removing acetyl groups from histones^65^. UNC5A with high expression is extremely sensitive to all three medications, and MCAM is highly sensitive to MS-275 and vorinostat. Studies showed that MCAM enhances the killing effect induced by vorinostat by inhibiting AKT (protein kinase B) pathway activation in cancer cells. The combination of MCAM and vorinostat can be used as a new treatment strategy for the more effective killing of cancer cells^66^. The highly-expressed NTN1 and DSCAM are extremely sensitive to MS-275. NEO1 is highly sensitive to vorinostat. These findings suggest that NTN1, DSCAM, and NEO1 are regulated by HDACs in renal cell carcinoma, thus providing new concepts for the use of HDACis in the treatment of renal cell carcinoma.

The structures of target proteins of NTN1 and its receptors obtained from the PDB database were docked with their related sensitive drugs to further screen potential drugs. We found that the drug with the highest binding capacity to 1 × 5I (The solution structure of the fourth fibronectin type III domain of human Neogenin) is VX-680 (Fig. 8c), a selective small molecule inhibitor of auroral kinase. It has been proved that it destroys movement and induces apoptosis in many tumor cell lines. The drug with the highest binding capacity to 4V2A (human Unc5A ectodomain) is GSK J4 (double inhibitor of H3K27me3/me2 demethylase JMJD3/KDM6B and UTX/KDM6A) (Fig. 8d).

### Therapeutic potential of NTN1 and its receptors in tumor immunotherapy

**Fig. 9:**
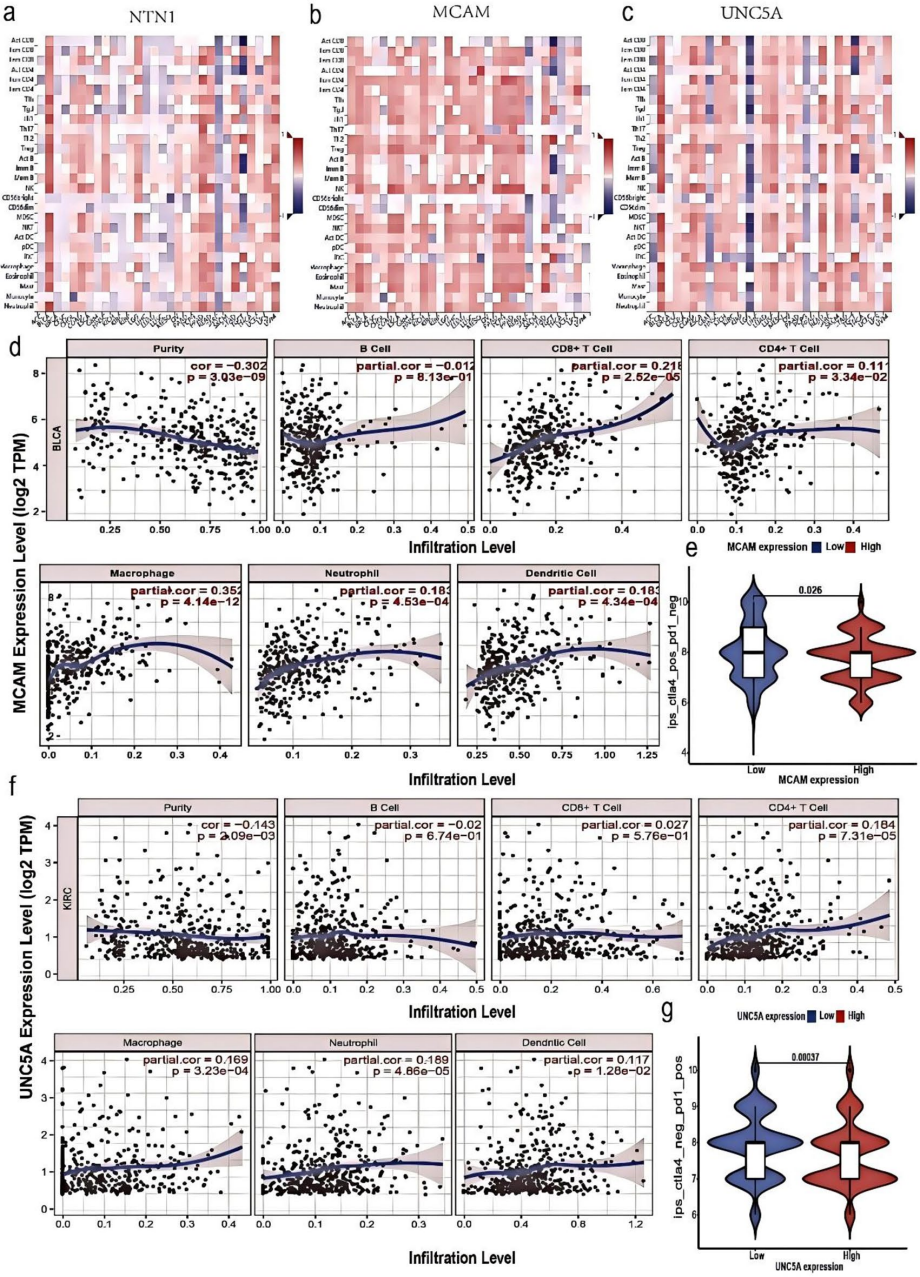
Correlation between the expression of NTN1 receptors and immune cell infiltration. (a) Relationship between the expression of NTN1 and 28 types of TILs across human cancers. (b) Relationship between the expression of MCAM and 28 types of TILs across human cancers. (c) Relationship between the expression of UNC5A and 28 types of TILs across human cancers. (d) The MCAM expression is negatively related to tumor purity and is correlated to dendritic cells, CD4 + T-cell, neutrophil, CD8 + T-cell, and macrophage in BLCA. (e) The relationship between the expression of MCAM and the score of immunotherapy in BLCA. (f) The UNC5A expression is negatively related to tumor purity and is correlated with dendritic cell, CD4 + T-cell, neutrophil, CD8 + T-cell, and macrophage in KIRC.(g) The relationship between the expression of UNC5A and the score of immunotherapy in KIRC.

We analyzed the correlation between the expression of NTN1 and its receptors and 28 types of tumor-infiltrating lymphocytes (TILs) using the TISIDB database. The most significant correlations were between NTN1, MCAM, UNC5A, UNC5B, and UNC5C and the 28 types of TILs. Figure 9a and b, and 9c show the relationship between the expression of NTN1, MCAM, and UNC5A and the 28 types of TILs in human cancer. As shown in Fig. 9a and b, and 9c, NTN1 and its receptors were correlated to the 28 types of TILs in many cancers, among which BLCA, KIRC, READ, and COAD were the most prominent.

We also analyzed the correlation between the expression of NTN1 and its receptors and 6 types of tumor-infiltrating immune cells using the TIMER database. As shown in Fig. 9d, the expression of MCAM in BLCA was associated with tumor purity (*r* = − 0.302), CD8 + T cells (*r* = 0.218), CD4 + T cells (*r* = 0.111), macrophages (*r* = 0.352), neutrophils (*r* = 0.183), and dendritic cells (*r* = 0.183). Furthermore, we downloaded and statistically analyzed MCAM and UNC5A expression, as well as immunotherapy score data from TCGA and The Cancer Immunome Atlas (TCIA) databases respectively. We found that patients with low MCAM expression had better immunotherapy effects in BLCA (Fig. 9e) (Table 1). These results suggested that MCAM may play an important role in the immune infiltration of BLCA. Figure 9f shows the correlation between UNC5A and the 6 types of tumor-infiltrating immune cells in KIRC. The expression of UNC5A was associated with tumor purity (*r* = − 0.143), CD4 + T cells (*r* = 0.184), macrophages (*r* = 0.169), neutrophils (*r* = 0.189), and dendritic cells (*r* = 0.117). As shown in Fig. 9g, the patients with low UNC5A expression had better immunotherapy effect in KIRC. Therefore, these results indicated that UNC5A may play an important role in the immune infiltration of KIRC.

**Table 1:** Core discovery summary.

Cancer types	Key genes	Molecular characteristics (mutation/ expression/ methylation)	Clinical association (prognosis/stage)	Potential mechanisms
Cutaneous melanoma (SKCM)	DSCAM, DCC	The mutation rate was highest (60.14%), with DSCAM hotspot mutation E368K	High mutations are associated with malignancy	Mutations affect Ig domain and enhance EMT activity
Head and neck squamous cell carcinoma (HNSC)	DCC, ROCK1	Silencing DCC methylation, forming ROCK1-DCC fusion gene	Fusion genes are associated with increased invasiveness	Disrupts the NTN1 binding domain and promotes cell migration
Renal clear cell carcinoma (KIRC)	UNC5D, NEO1	Upregulation of UNC5D methylation (positively correlated with pathological stage)	Low UNC5D expression predicts poor prognosis	Methylation inhibits UNC5D and removes EMT inhibition
Urinary bladder urothelial cancer (BLCA)	MCAM, UNC5B	High expression of MCAM and UNC5B are associated with poor prognosis	Immunotherapy effect is better in patients with low MCAM expression	MCAM regulates the immune microenvironment through macrophage infiltration

## Discussion

Netrin1 receptors play important biological functions in neurodevelopment and tumorigenesis. However, systematic research on the regulatory mechanisms of its expression characteristics and potential cancer biological functions in pan-cancer is lacking. In the present study, we performed a multi-dimensional analysis of netrin1 receptors using computational biology methods combined with the multigroup data of 10,437 subjects obtained from TCGA. We found that netrin1 and its receptors showed stereotypical genetic changes in tumor-suppressor genes or oncogenes. Promoter methylation and miRNA-mediated post-transcriptional inhibition may represent major regulatory mechanisms. EMT promotion was the standard cellular function of netrin1 and its receptors. Our results suggest that netrin1 receptors are potential tumor markers and therapeutic targets with high transformational medical values.

Our study is the first to reveal significant tumor-related genetic changes in netrin1 receptors from a pan-cancer multi-histological perspective(The core findings are detailed in Table 1). First, the overall mutation rate of netrin1 receptors in the pan-cancer survey was 7%–35% (Fig. 2e), in which DSCAM (35%), DCC (33%), and UNC5D (20%) showed higher mutation rates and these mutations occurred in highly malignant endometrial cancer and skin melanoma. Moreover, we found that the mutations in netrin1 and its receptors were significantly enriched in critical functional regions (including ligand–receptor binding domain and death domain) and functional sites (protein post-translational modification sites and enzyme cleavage sites), suggesting the potential biological function of these mutations in various cancers. Second, except for MCAM and UNC5B, 6 netrin1 receptors formed fusion genes in many types of solid tumors. Previous studies reported that UNC5C expression was downregulated because of the fusion gene with SNX9 in prostate cancer^67^. However, our pan-cancer analysis showed that among the members of the UNC5 family, UNC5D formed the most fusion genes, NEO1 formed fusion genes in various endocrine-associated cancers (OV, BRCA, and PRAD), and some metabolic enzymes with a strong upstream promoter and oncogene functions formed fusion genes with diverse netrin1 receptors. These findings suggest that the formation mechanism and biological cancer function of the netrin receptor fusion gene should be explored in the future. Although transcriptomic data relying on databases such as TCGA cover gene expression, mutation and methylation information in multiple cancer species, it lacks expression verification at the protein level and may not fully reflect the actual functional state of the gene. Subsequent research will focus on supplementing evidence of protein levels through laboratory verification and clarifying specific molecular mechanisms in conjunction with functional experiments to enhance the reliability of research conclusions and clinical transformation value.

We also identified multiple regulatory mechanisms of netrin1 and its receptors at the epigenetic and transcriptional levels. Many studies reported that UNC5 family members and DCC are downregulated at the single gene level because of promoter hypermethylation in colorectal cancer, gastric cancer, renal cell carcinoma, lung cancer, and other solid tumors. Consistent with these observations, our pan-cancer analysis results suggest that UNC5D is the most methylated member of the UNC5 family. Moreover, DSCAM, which participates in the development of the central nervous system and related diseases, showed hypermethylation in various solid tumors. Based on pan-cancer data analysis, we found that netrin1 and its receptors showed coexpression patterns in many tumors at the multigene level. In some solid tumors, including renal clear cell carcinoma, the expressions of netrin1 and its receptors were correlated with prognosis. These results suggest the need for exploring the synergistic mechanism of netrin1 and its receptors in pan-cancer. Moreover, consistent with the co-methylation of netrin1 and its receptors found in breast cancer, NTN1 showed a co-methylation pattern with different receptors such as UNC5D in colorectal cancer, endometrial cancer, lung cancer, and other tumors. The corresponding coregulation mode also appears in EZH2- or HDAC2-mediated histone modification. In particular, miRNA-mediated post-transcriptional inhibition is another important probable mechanism that regulates the expression of netrin-1 and its receptors. Our findings suggest that 30 miRNAs, including let-7 and miR-130, inhibit the mRNA expression of netrin-1 and its receptors in many solid tumors. On the contrary, some studies have confirmed that TP53 regulates the expression of NTN1 and UNC5 family members. We found that both TP53 and TP63 may regulate the homologous gene NEO1 of DCC and that it was relatively sensitive to proapoptotic medications. The synergistic regulation of expression via probable and transcriptional regulatory mechanisms has scope in future therapeutic intervention studies on the expression of netrin1 and its receptors. Netrin1 is an axon-oriented molecule with strong chemotactic effects on axon guidance, cell migration, and angiogenesis. Consistent with these observations, in our pan-cancer study, we found that netrin1 and its receptors promote EMT, The NTN1 receptor MCAM activates Wnt/β-catenin through the integrin/ FAK pathway and upregulates EMTs such as Snail^13^. UNC5D is under-expressed in renal cell carcinoma (KIRC) due to promoter methylation, which removes EMT inhibition, and co-methylation with NTN1 cooperates with EZH2 to drive metastasis^40,46^, suggesting that abnormal expression/mutation of NTN1 and its receptor is significantly associated with activation of the EMT pathway, suggesting that it may participate in the regulation of tumor progression. Further research on factors interfering with the signal pathways mediated by netrin1 and its receptors may reduce the risk of tumor metastasis, Which provides a basis for targeting MCAM or combining HDAC inhibitors (such as vorinostat) to intervene in metastasis^47^.The essence of the differential effects of DCC and UNC5B is their “receptor-dependent” characteristics and signaling pathway bias: DCC is more likely to promote tumor progression through proliferation and migration pathways, while UNC5B affects tumor outcome through cell cycle regulation, apoptosis signals and immune microenvironment regulation^3,8^. Subsequent studies can combine the differences between the two in methylation, fusion genes (such as DCC-ROCK1) and miRNA regulation (such as hsa-miR-4726- 5p inhibition of UNC5B) to further verify its specificity as a therapeutic target.Currently, intervention in the netrin-1 pathway (such as neutralizing antibodies against netrin-1) has been explored clinically, and this study further suggests that there are differences in the expression, mutation and regulatory mechanisms of netrin-1 receptor in different cancer species (such as high-frequency mutations of DSCAM in SKCM, UNC5D methylation in KIRC), which requires clinical research to “precise policies” based on the receptor characteristics of tumor subtypes. For example, for renal clear cell carcinoma driven by UNC5D methylation, a combination protocol of “HDAC inhibitor (restoring UNC5D expression) + netrin-1 antibody” can be explored to simultaneously inhibit tumor proliferation and immune evasion. This study provides specific targets and sensitivity basis for the development of netrin-1 receptor-related drugs, and also provides experimental support for marker screening and joint strategy design for immunotherapy, which can further promote the netrin-1 pathway in clinical research. Precise application.

Another significant result of this study suggested that NTN1 and its receptors can be used as potential targets for tumor immunotherapy. Previous studies have shown that NTN1 and its receptors are related to the immune system; e.g., Netrin-1 acting on its homologous receptor UNC5B inhibited CC chemokine-induced immune cell migration^68^ and the interaction between Netrin-1 and Neo1 increased the chemotactic movement of CD4 + T cells and promoted cell infiltration associated with acute inflammation^69^. By the comprehensive analysis of tumor–immune system interactions, we found that the difference in UNC5B expression was significant in urothelial carcinoma and melanoma immunotherapy, and the difference in NTN1 expression was also significant in melanoma immunotherapy. Furthermore, we found that the expression of NTN1 and its receptors in several cancers, including BLCA, KIRC, READ, and COAD, was closely associated with tumor-infiltrating immune cells such as CD4 + T cells, CD8 + T cells, macrophages, neutrophils, and dendritic cells. For example, CD8 + T cells, which are related to MCAM expression, were associated with BLCA prognosis, whereas UNC5A expression in KIRC was positively correlated with many TIL types, such as Tcm_CD4 + T cells. These findings suggest that NTN1 and its receptors have a potential and expectant correlation with immune infiltration and solid tumor survival (e.g., BLCA, KIRC, READ, and COAD). Further statistical analysis showed that the immunotherapy score of patients with low MCAM expression was relatively higher in BLCA and the immunotherapy score of patients with low UNC5A expression was higher in KIRC. All these results provide a basis for future studies on the role of NTN1 and its receptors in immunotherapy. The netrin-1 receptor affects tumor progression through a multi-dimensional mechanism of “immune checkpoint regulation-immune cell recruitment-inflammatory factor secretion-EMT/ECM remodeling”. Its role is receptor-specific: MCAM is biased towards immunosuppression and EMT activation, UNC5B/5D balances proliferation and metastasis through apoptosis signaling and methylation regulation, and DCC/DSCAM is more involved in immune cell chemotaxis and ECM degradation^12,13^. Combined with the data on methylation, fusion genes (such as DCC-ROCK1) and drug sensitivity (such as MCAM and vorinostat) in the paper, the value of these mechanisms in clinical transformation can be further verified and the joint strategy of “receptor targeting + immunotherapy” provides theoretical support. However, further studies should be designed to confirm these correlations and potential mechanisms.Because the analysis conducted through the database may be biased, the sample collection process is not completely random, and factors such as region and ethnicity will affect the representativeness of the samples, resulting in the study results not accurately reflecting the true situation of all cancer patients. Therefore, the potential molecular mechanism still needs to be further confirmed by in vivo and in vitro experiments.

## Conclusion

In short, our findings help explain the tumor-related molecular biological characteristics of netrin1 and its receptors from multiple dimensions on the basis of multidimensional data including genetic biology, epigenetics, and pharmacogenomics. Our pan-cancer study results revealed significant common genetic changes in netrin1 and its receptors, the synergistic regulatory mechanisms of ligand receptors, the carcinogenic signal pathway, potential drug and their correlation with immune cell infiltration. Although the underlying molecular mechanisms are yet to be confirmed by in vivo and in vitro experiments, our results reveal the transformation potential of netrin1-related receptors.

## Supplementary Information

Below is the link to the electronic supplementary material.

Supplementary Material 1

Supplementary Material 2

Supplementary Material 3

Supplementary Material 4

## Data Availability

The datasets generated during and analyzed during the current study are available in Uniprot database (https://www.uniprot.org/), Cbioportal database (http://www.cbioportal.org/), TCGAAA database (http://52.25.87.215/TCGAA/), VarCards database (http://varcards.biols.ac.cn/), UET database (http://mammoth.bcm.tmc.edu/uet/), CCLE database (https://portals.broadinstitute.org/ccle/about), OncoPrinter database (http://www.cbioportal.org/oncoprinter), Mutation Mapper database (http://www.cbioportal.org/mutation\_mapper), Swissmodeldatabase (https://swissmodel.expasy.org/), dbPTM database (http://dbptm.mbc.nctu.edu.tw/), TCGA fusion gene database (http://www.tumorfusions.org/), ENSEMBL database(http://asia.ensembl.org/), CCDS database (https://www.ncbi.nlm.nih.gov/CCDS), GSCA database (http://bioinfo.life.hust.edu.cn/web/GSCALite/), Kaplan–Meier Plotter database (http://kmplot.com/analysis/), LinkedOmics database (http://www.linkedomics.org/Login.php), GSCALite database (http://bioinfo.life.hust.edu.cn), SMART database (http://www.bioinfo-zs.com/smartapp/), miRWalk database (http://mirwalk.umm.uni-heidelberg.de/), STARBASE v3.0 database (http://starbase.sysu.edu.cn/), STRING database(https://string-db.org/), PharmacoDB database (https://pharmacodb.pmgenomics.ca/), TCIA database(https://tcia.at/), TIMER database (https://cistrome. shinyapps. io/timer/). The original contributions presented in the study are included in the article/Supplementary Material. Further inquiries can be directed to the corresponding author.
