# Supplementary material for: Pan-cancer analysis reveals netrin-1 receptors as potential tumor biomarkers and immune-related therapeutic targets
Source: Sci Rep. 2025 Dec 17;15:44015. doi: 10.1038/s41598-025-28437-0 (PMC12711908; doi:10.1038/s41598-025-28437-0)
Supplement: Supplementary file 4 — Supplementary Material 4 [file 41598_2025_28437_MOESM4_ESM.doc]

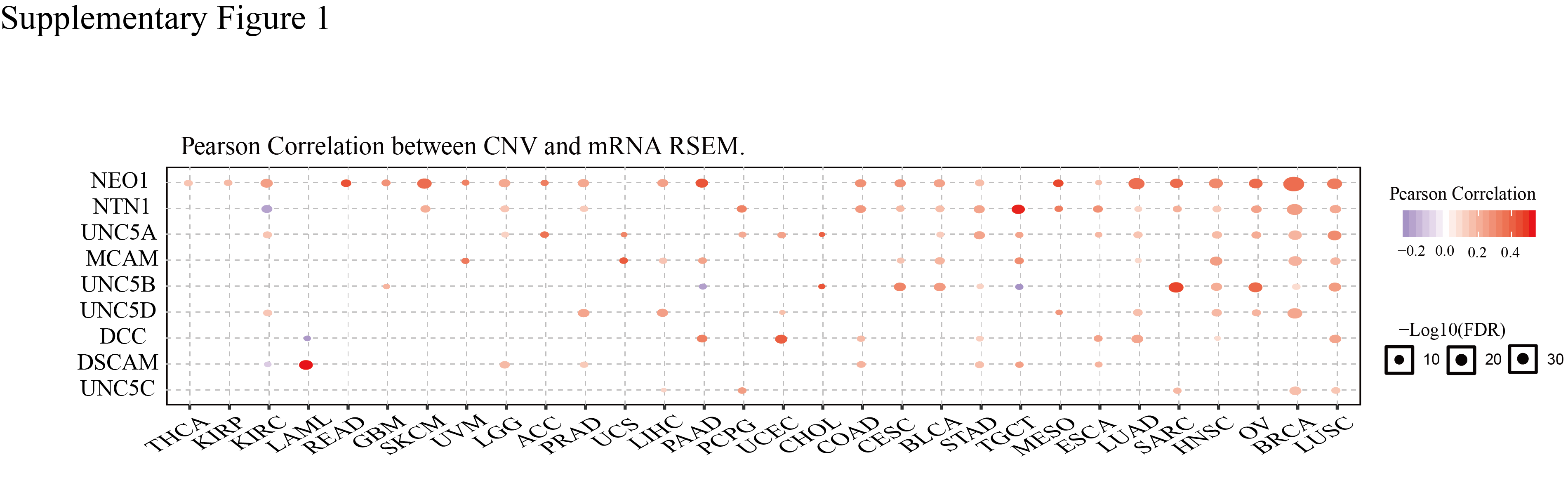
Supplementary Fig 1. Correlation between CNV and mRNA expression of NTN1 and its receptors. This figure illustrates the correlation between copy number variations (CNV) and mRNA expression levels of NTN1 and its receptors (DCC, UNC5A-D, NEO1, DSCAM, MCAM) across 33 TCGA cancer types.


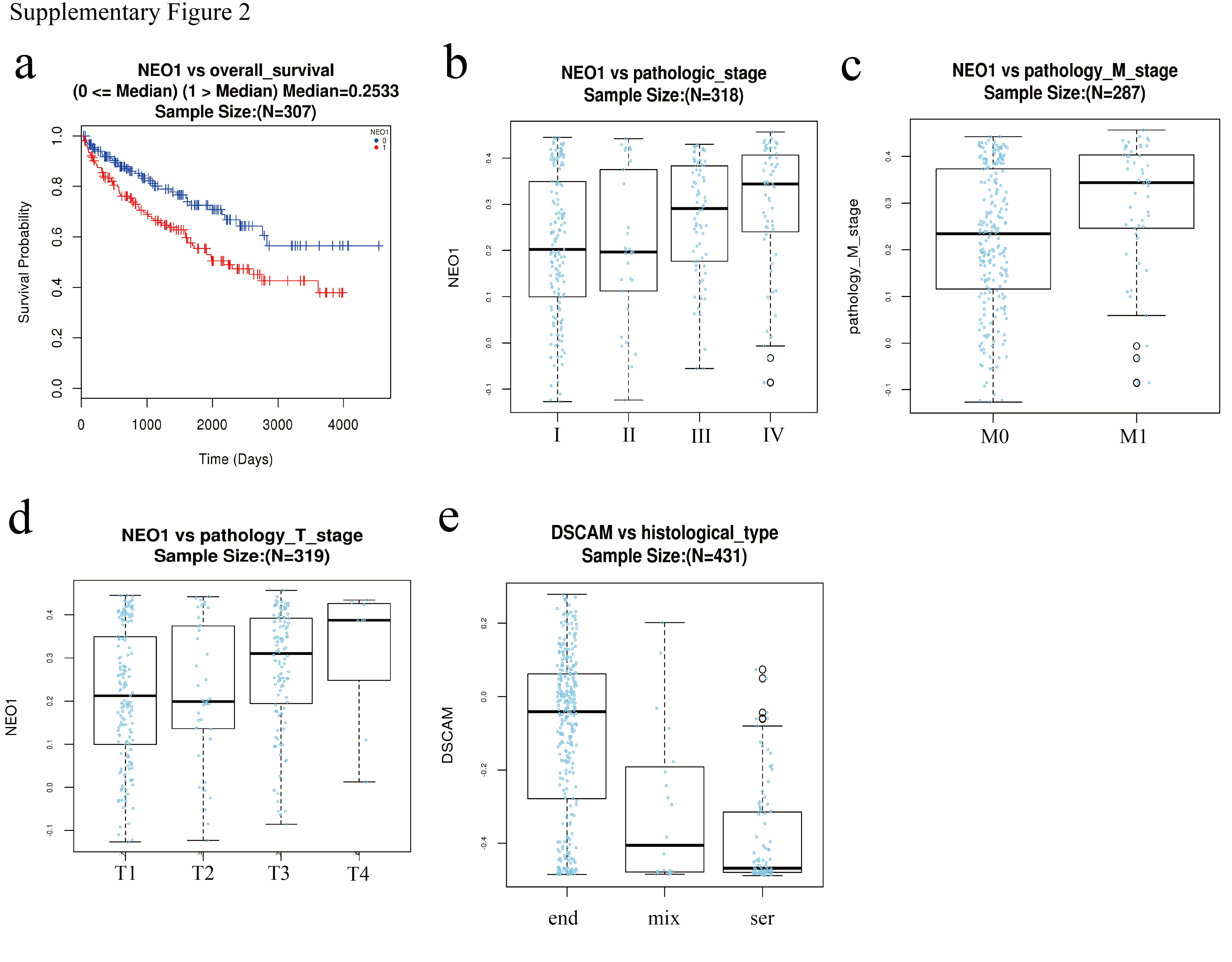


Supplementary Fig 2. Methylation and clinical correlation analysis of NEO1 and DSCAM

This figure presents the methylation status of NEO1 and DScAM and their clinical correlations in specific cancers.


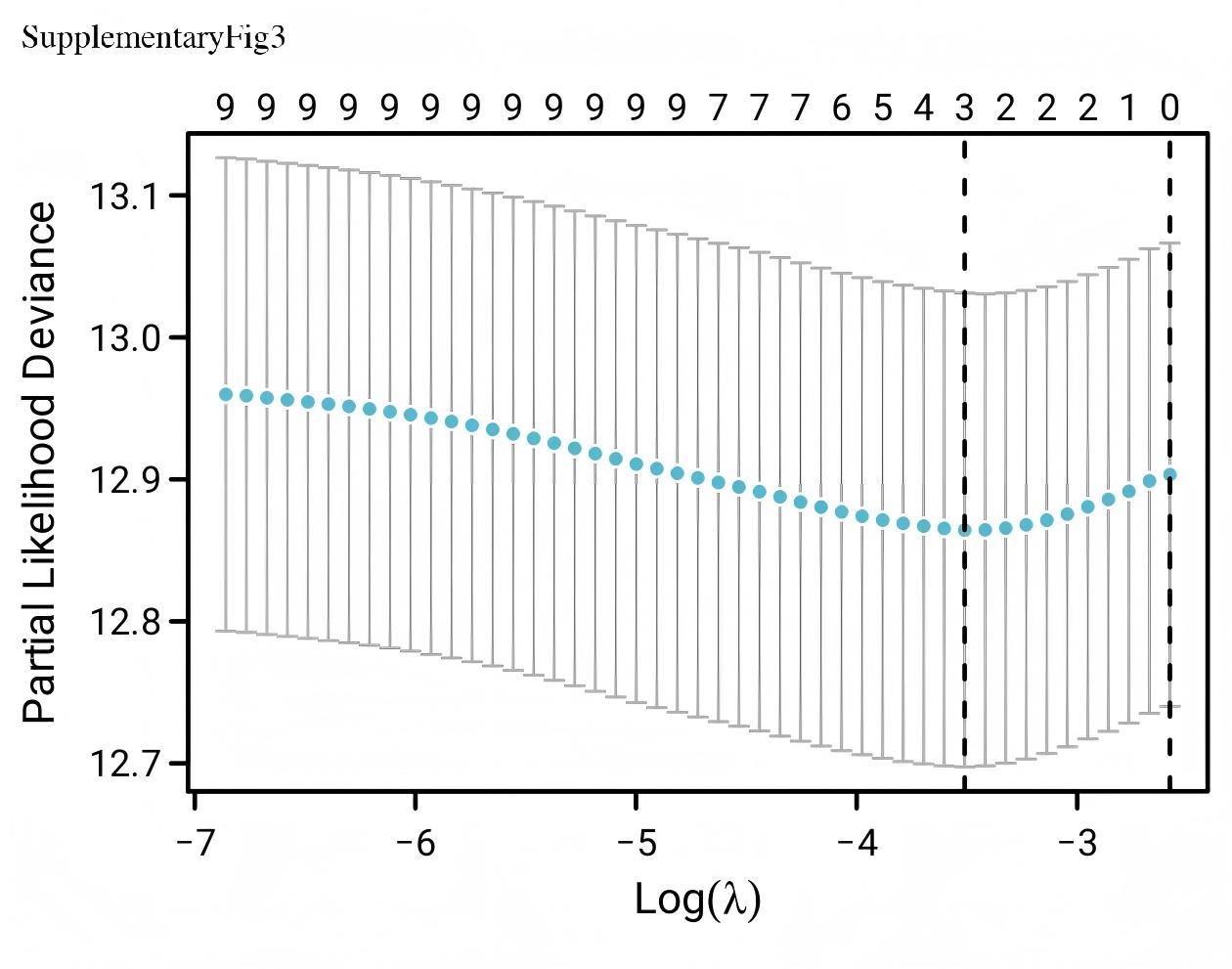


Supplementary Figure 3.Lasso-penalized Cox analysis for prognostic index construction


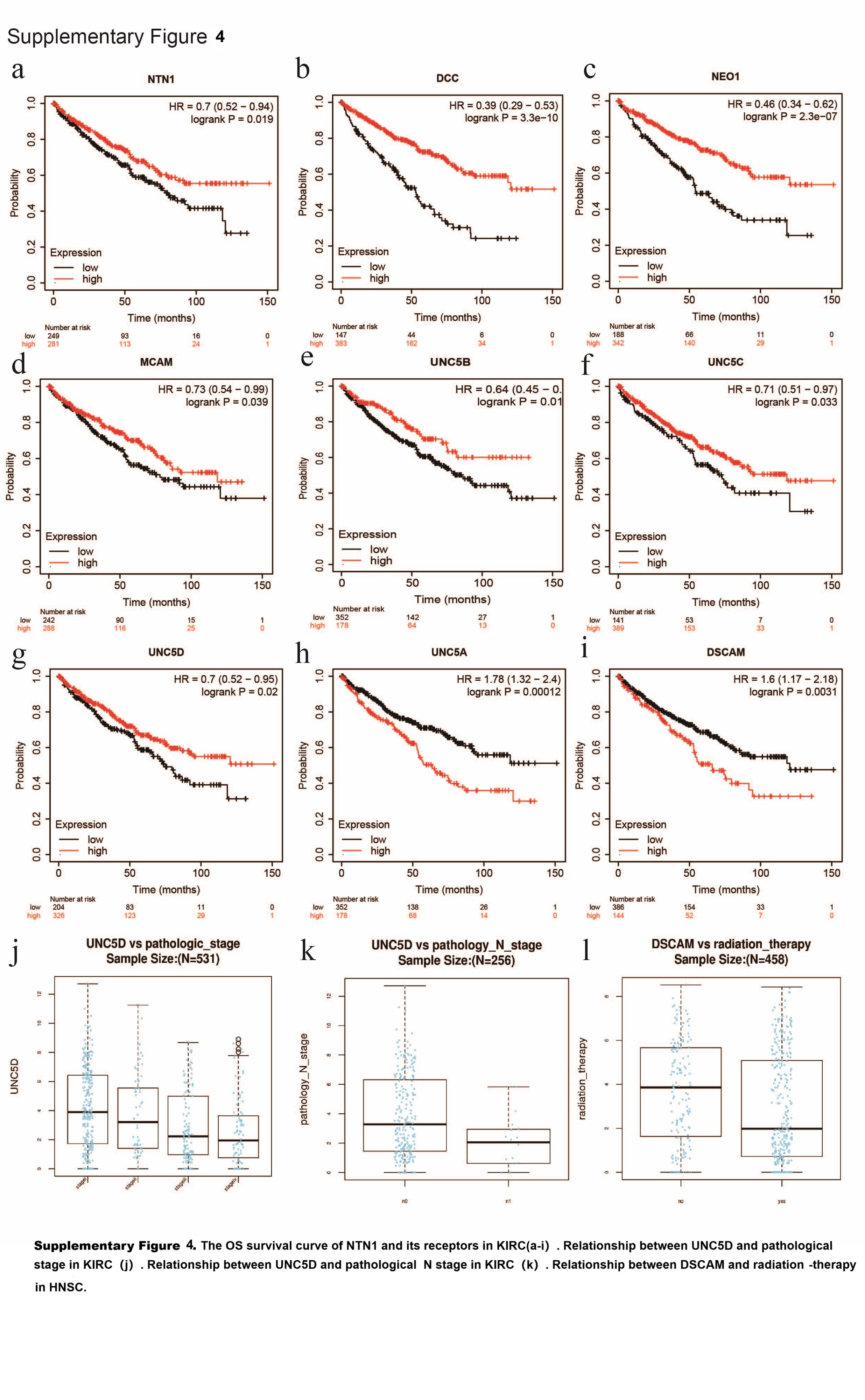


Supplementary Figure 4. The OS survival curve of NTN1 and its receptors in KIRC(a-i) . Relationship between UNC5D and pathologicalstage in KIRC (i) . Relationship between UNC5D and pathological N stage in KIRC (k) . Relationship between DSCAM and radiation -therapy in HNSC.
